# Study protocol: short against long antibiotic therapy for infected orthopedic sites — the randomized-controlled SALATIO trials

**DOI:** 10.1186/s13063-023-07141-2

**Published:** 2023-02-18

**Authors:** Ilker Uçkay, Stephan Wirth, Björn Zörner, Sandro Fucentese, Karl Wieser, Andreas Schweizer, Daniel Müller, Patrick Zingg, Mazda Farshad

**Affiliations:** 1 Department of Orthopedic Surgery, Balgrist University Hospital, University of Zurich, Zurich, Switzerland; 2 Unit for Clinical and Applied Research, Balgrist University Hospital, University of Zurich, Zurich, Switzerland; 3 Infectiology, Balgrist University Hospital, University of Zurich, Zurich, Switzerland; 4 Spinal Cord Injury Center, Balgrist University Hospital, University of Zurich, Zurich, Switzerland; 5 Hand Surgery, Balgrist University Hospital, University of Zurich, Zurich, Switzerland; 6 Medical Direction, Balgrist University Hospital, University of Zurich, Zurich, Switzerland

**Keywords:** Orthopedic infections, Osteomyelitis, Surgical debridement, Antibiotic duration, Remission, Adverse events

## Abstract

**Background:**

Few studies address the appropriate duration of post-surgical antibiotic therapy for orthopedic infections; with or without infected residual implants. We perform two similar randomized-clinical trials (RCT) to reduce the antibiotic use and associated adverse events.

**Methods:**

Two unblinded RCTs in adult patients (non-inferiority with a margin of 10%, a power of 80%) with the primary outcomes “remission” and “microbiologically-identical recurrences” after a combined surgical and antibiotic therapy. The main secondary outcome is antibiotic-related adverse events. The RCTs allocate the participants between 3 vs. 6 weeks of post-surgical systemic antibiotic therapy for implant-free infections and between 6 vs. 12 weeks for residual implant-related infections. We need a total of 280 episodes (randomization schemes 1:1) with a minimal follow-up of 12 months. We perform two interim analyses starting approximately after 1 and 2 years. The study approximatively lasts 3 years.

**Discussion:**

Both parallel RCTs will enable to prescribe less antibiotics for future orthopedic infections in adult patients.

**Trial registration:**

ClinicalTrial.gov NCT05499481. Registered on 12 August 2022.

Protocol version: 2 (19 May 2022)

**Supplementary Information:**

The online version contains supplementary material available at 10.1186/s13063-023-07141-2.

## Background and rationale

The optimum duration of postoperative, systemic antibiotic treatment for orthopedic infections, with or without implant removal, or immune suppression, is unknown [1, 2].

Because of the potential for poor outcomes, many clinicians treat bone and implant infections with a long course of antibiotic therapy. While there are few data supporting the need for prolonged therapy for bone and implant infections, such an approach certainly increases the risk of side effects, development of antibiotic resistance, and costs [3]. Moreover, with only a few exceptions, the recommended durations do not consider the various surgical approaches or the causative microorganisms. The only substantial adjustment regarding the duration of antimicrobial treatment is the presence, or absence, of an infected implant kept in place. Practically, if there is an implant in place, clinicians simply double the administration time; e.g., from 6 weeks of antibiotic therapy to 12 weeks. No one knows why there should be exactly a doubling in duration, and not, let’s say, a 1.5-times longer treatment. It is just tradition that has not been revoked.

We can do better. In a world where the emerging problem of antibiotic resistance gains momentum and policymakers and Infectious Diseases Physicians perform many prospective trials in all sorts of human infections to improve an antibiotic stewardship and reduce the unnecessary burden of antibiotic consumption, we do not understand why these efforts should not be made for the management of orthopedic infections. The study team of the SALATIO trials believes in antibiotic stewardship in orthopedic surgery [1–12], provided that the surgical debridement is performed according to the state-of-the-art [3, 7, 10].

Retrospective studies suggest that a maximum duration of 6 weeks is not inferior to longer durations [1–5, 13, 14]; even if the infected material is kept in place [1, 3, 13, 14]. Prospective-randomized trials (RCT) reveal the same results, only the shorter antibiotic arm is arbitrarily fixed to 6 weeks. Most RCTs randomize between 6 and 12 weeks. In these prospective studies [6–10], 6 or 8 weeks of systemic antibiotics are not inferior [10–12] to the current 12 weeks during the DAIR procedure (debridement, antibiotic, and implant retention) for arthroplasty infections [1, 3, 12], or during a one-stage exchange [2, 4]. The only exception is one single RCT suggesting a better outcome in favor of 12 weeks among arthroplasty infections undergoing the DAIR procedure [15].

We are convinced that in orthopedic bone and implant infections, the shorter systemic antibiotic durations are as good, or as bad, as longer durations after surgical debridement. The duration of postsurgical antibiotic prescription is probably less important than other clinical variables such as the surgical approach [4, 7] or the patient’s compliance [3, 16, 17]. Almost all retrospective studies, deny a decisive role of the total antibiotic duration in terms of clinical failures, remission, or recurrences of infections. At the same, we have only rudimentary data advocating for lesser durations than for the arbitrary level of 6 weeks, especially in implant-free infections. The SALATIO trials are specific RCTs scheduled to fill this gap in the scientific literature. In contrast, the trials do not investigate the management of soft tissue infections or native joint septic arthritis cases [18], which we consider as very different clinical entities compared to bone. Likewise, we do not investigate the reduction of systemic antibiotic use concomitantly with a local, intraosseous antibiotic release, which is currently studied by other research groups [19]. Finally, we adapt our study documents to the SPIRIT criteria [20–22] that we consider as state-of-the-art in RCT reporting.

## Methods

### Study setting

The Balgrist University Hospital in Zurich is a tertiary, referral center for orthopedic surgery (including for orthopedic infections) and is affiliated to the University of Zurich. It resumes a multi-disciplinary team composed of orthopedic surgeons, internist physicians, specialized nurses and physiotherapists, musculoskeletal expert radiologists, and infectious diseases physicians who are specialized in orthopedic infections. Moreover, this team is supported by a research campus (Balgrist Campus) with BioBanking and a Unit for Clinical and Applied Research with experience in biostatistics and investigative designs (www.balgrist.ch). The SALATIO trials start at the Balgrist, but are expandable to other (international) centers. Supplementary File 1 is the original Protocol. Supplementary File 2 is a model Consent Form in English and adapted to SPIRIT criteria. The original consent form is in the German language.

### Study objectives and outcomes

We investigate the possibility to reduce the post-debridement antibiotic duration in almost all strata of orthopedic infections among adult patients. We conduct two RCTs embedded in the ongoing register of orthopedic infections. The first RCT concerns patients with a retention of the infected implant (or its change) during the first surgical debridement. Here, we evaluate if 6 weeks of systemic and targeted antibiotic therapy postoperatively is not inferior to 12 weeks (non-inferiority trial) after a follow-up of 12 months. For infections with complete implant removal (or osteomyelitis with external fixation only), the second RCT investigates if 3 weeks of post-surgical antibiotic therapy is not inferior to 6 weeks after a follow-up of 12 months. The primary outcomes are always the remission at 1 year postoperatively and the proportion of recurrent infections due to the same pathogens as in the index episode. The secondary outcomes are adverse events, with an emphasis on antibiotic-related events during the intervention and the length of hospital stay in an acute care setting. We do not evaluate costs.

### Definitions and eligibility criteria for participants

An “orthopedic infection” is the concordant microbiological evidence of bacteria in at least two deep intraoperative tissue samples together with radiological (osteomyelitis, collections, inflammation) and/or clinical evidence of infection (pus, discharge, sinus tracts, rubor, color, pain). Histological proof is facultative for this study. Implants are defined as any foreign material; except for allografts, transient wires, or fixator pins outside of the infected bone. Remission of infection is the absence of clinical, and/or radiological, and/or laboratory signs of the (former) infection after the minimal follow-up time of 12 months. Inversely, a clinical failure is any problem leading to unplanned revision surgeries, including for persistent infection during antibiotic therapy, non-infectious reasons (i.e., seroma, hematoma, implant failure, wound dehiscence, fractures), or new infection at the same site. A microbiological recurrence is a recurrence with the same pathogen(s) after the end of the antibiotic therapy.

### Interventions and study conduct

**Fig. 1:**
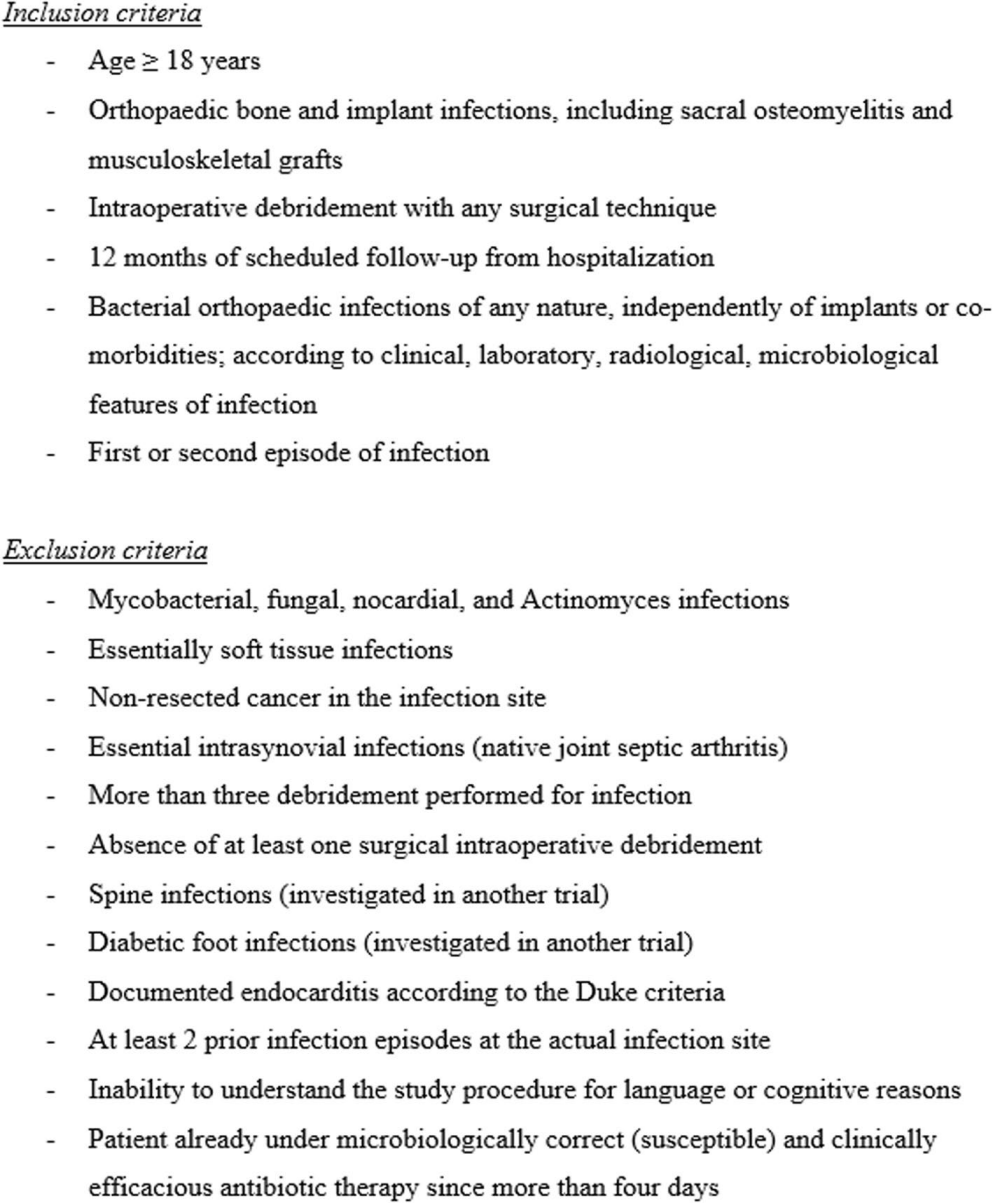
Study criteria

**Fig. 2:**
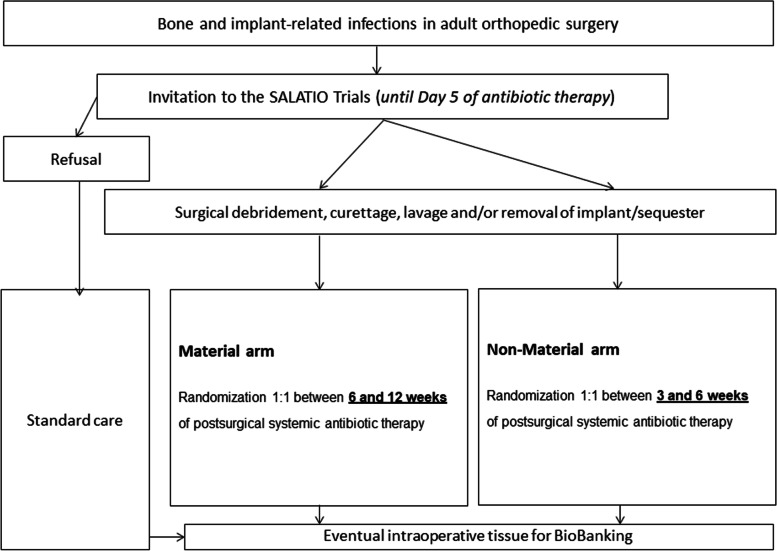
Main study flowchart

When surgeons, infectious diseases physicians, and internists are consulted for orthopedic infections, they will screen the patients. If inclusion and no exclusion criteria are met, the patient will be informed about the study by the investigators. Upon his/her written consent, we perform two parallel RCTs, depending on the presence of osteosynthesis material at enrolment: SALATIO 1: Infected implant not removed (or new material inserted): Randomization 6 vs. 12 weeks (+/− 5 days) of total antibiotic therapy counted since the first debridement for infection. SALATIO 2: Infected implant without residual material (definitive removal or within the interval of a two-stage exchange): Randomization 3 vs. 6 weeks (+/− 5 days) of total antibiotic therapy counted since the first debridement for infection. Figure 1 resumes the formal study criteria and Fig. 2 the main study flowchart. There will be no blinding of persons and no placebos.

At study entry, we assess the following variables:

- Patient’s characteristics: age, sex, known immune suppression (diabetes, renal dialysis, cirrhosis, pregnancy, medicamentous immunosuppression, untreated HIV disease, agranulocytosis, active cancer), American Society of Anesthesiologists (ASA)-Score
- Surgery and infection data: number and type of surgeries for the actual problem, agent, dose, and duration of pre-surgical antibiotic therapy, local antibiotics used in the bone, cell count (if any), initial serum C-reactive protein (CRP) level, presence of initial bacteremia, implants, microbiological results, histology (facultative)
- Treatment and outcomes: number of surgeries to treat infection, total duration of antibiotic therapy, duration, agent, and dose of intravenous and oral antibiotic therapy, wound healing problems, presence and duration of vacuum-assisted negative pressure therapy, all adverse events, clinical or microbiological recurrence, date and reasons for re-hospitalization and re-treatment, follow-up data, fatalities
- Administrative data: total hospitalization length, eventual BioBanking of infected tissues

**Fig. 3:**
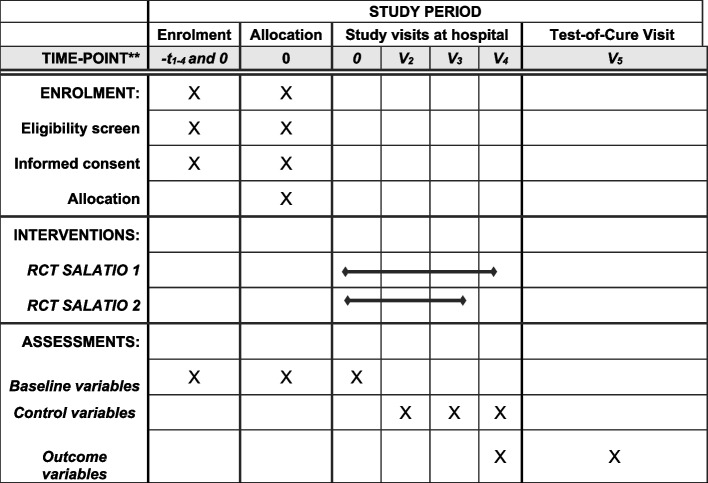
SPIRIT flowchart of the enrolments and assessments during the randomized-controlled trials

After randomization, the study participants will be actively followed-up for a minimum of 12 months. We will equally review the medical charts of all patients to seek for unscheduled visits. This “passive follow-up” can reach up to 4 years and terminates at the date of database closure. The scheduled study visits take place as follows (Fig. 3).

- Visit 1 - enrollment (day 1)
- Visit 2 - day 21 (+/− 5 days)
- Visit 3 - day 42 (+/− 5 days) (standard surgical controls in our hospital)
- Visit 4 - day 84 (+/− 5 days) (if not in the short antibiotic arm)

End of treatment (EOT) visit - day 21, 42, or 84 (+/− 5 days) (only if still receiving treatment after visit 2). Test-of-cure (TOC; visit 5) − 12 months (+/− 2 months).

During the study visits, we assess the history, adverse events, and the surgical (functional) status. We examine the patients according to the orthopedic standard and add supplementary laboratory exams and radiology only if clinically indicated. Only visit 2 can be by telephone alone. If the patient is currently hospitalized for rehabilitation under medical supervision, the visits can be replaced by the medical assessment during the reeducation/rehabilitation. A microbiologically effective antibiotic therapy beyond 96 h prior to screening is not permitted. However, a 72-h window before debridement is permitted, independently of the duration of prior antibiotic administration. If the patient requires a new antibiotic agent based on microbiological results; independently of the duration of prior ineffective antibiotic therapy, there are no minimal windows or maximal pre-debridement antibiotic durations. Initially, antibiotic therapy is either empiric or targeted to the results of preoperative information. After 2–4 days, antibiotic therapy becomes targeted to the susceptibility profile. The choice of the agent, and its administration route (oral or parenteral), is at the discretion of the treating clinicians. Nonetheless, for both RCTs, and to achieve a minimal homogeneity, we establish a list of “allowed antibiotics” (Table 1). The investigators must choose among them, unless the pathogens are very special. We will not test new indications for antibiotic therapy. Only the duration of the treatment will be determined. We avoid placebos, topical antibiotics on the skin, and antiseptics, except for the pre-incisional skin preparation. Likewise, anesthesiologists remain free to administer the routine perioperative prophylaxis (cefuroxime, vancomycin, or clindamycin), if they judge it indicated. The Pharmacy of the Balgrist will supply all antibiotics without study-specific packing or labeling. These trials, all antibiotics and surgeries, have no specific relations to pregnant or breastfeeding women and their children. However, the investigators will avoid antibiotics that are not liberated for pregnant or breast-feeding women, according to the Swiss Compendium (www.compendium.ch).

**Table 1:** List of allowed antibiotic treatments (empirical or targeted)

Antibiotic	Allowed dosing regimens	Allowed total daily dose range
Levofloxacin PO	750 mg q.24h or 500 mg q.12h	750 to 1500 mg
Ciprofloxacin PO	750 mg q.24h or 500 mg q.12h	750 to 1500 mg
Amoxicillin/clavulanate IV	1000/200 mg q.8h	2000/400 mg to 3000/600 mg
Cefuroxim IV	1500 mg q.8h	4500 mg
Ceftriaxon IV	2000 mg q.24 h	2000 mg
Co-trimoxazole PO	960 mg q.12h or q.8h	1920 mg to 2880 mg
Clindamycin PO	300 mg or 450 mg q.6h	1200 mg to 1800 mg
Doxycyclin PO	100 mg q.12h	200 mg
Minocyclin PO	100 mg q.12h	200 mg
Linezolid PO	600 mg q.12h	1200 mg
Metronidazole PO	500 mg q.8h	1500 mg
Vancomycin IV	15 mg/kg q.12h	According to serum through levels
Daptomycin IV	8 mg/kg/day	6–10 mg/kg/day
Cefepime IV	2000 mg q.12h	4000 mg
Imipenem IV	500 mg or 1000 mg or q.8h or q.6h	2000 mg to 3000 mg
Meropenem IV	2000 mg q.8h	1000 mg q.8h
Piperacillin/tazobactam IV	4000/500 mg q.8h	12,000/15,000 mg (12 g/1.5 g)

*PO* oral therapy, *IV* intravenous therapy

### Adherence to the study protocol and promotion of participant’s follow-up

Principally, the study protocol and all patients’ follow-ups are oriented to the usual surgical procedures of our clinic. All study-relevant data are part of normal clinical management. The study visits correspond to the usual surgical controls. Only the duration of the postsurgical antibiotic course is new and determined by the study allocation. Moreover, the Principal Investigator is the main Infectious Diseases Physician and all surgeons in the study team have a long experience in orthopedic infections. In our small region, the general practitioners, or other colleagues in other hospitals, do not change a study regimen without contacting us. This is also what we do with the study of patients of other hospitals. We contact the clinician in charge. If a patient does appear to the clinical controls, the secretary calls him/her by phone and/or by e-mail. For all these reasons, we think that the possibility of a major protocol violation or a loss to follow-up is unlikely when conducting our SALATIO trials.

### Risks for the participants

All patients can witness adverse events related to surgical and anaesthesiologic procedures and antibiotic administrations, which, however, are related to the therapy itself, and not to the protocol. A theoretical risk could be a higher incidence of microbiological recurrences in the short-duration arms. In contrast, future patients might benefit from shorter therapies, shorter hospital stays, and a reduced risk of adverse events. Our project is a contribution to the professional efforts of antibiotic stewardship that is gaining momentum worldwide.

### Allocation and compliance with randomization

After written informed consent has been given (up to 5 days after surgery), participants will be randomized with a 1:1 ratio by predefined study nurses drawing a sealed envelope that contains a randomization card. Study nurses with experience in clinical trials will generate these cards. They will monitor their use and keep them locked in another office outside of the clinic. Upon randomization, the study team asks the study nurses to randomize. Then, the study nurse randomizes within 24 h without knowing the identity of the new study entry. Importantly, these nurses are separated from the treating clinicians. The investigators will inform the patients and the treating clinicians about the randomization arm. After the assignment, we purposely lack any firm criteria for discontinuing or modifying the allocated interventions for a given trial study participant. Principally, the patient may withdraw his/her consent or is dropped out by the study team. Upon the detection of unintended protocol violations, the Principal Investigators decide if the patient continues the SALATIO trial in the intention-to-Treat population (by exclusion from the per-protocol population), or is dropped out. The patient never switches from one allocation arm to the other.

### Monitoring

The Unit for Clinical and Applied Research will assign an independent monitor (with experience in RCTs) to the study. All patient files, notes, and copies of laboratory and medical test results must be available for monitoring. The monitor will verify all, or a part of the Case Report Forms (CRF), data, and written informed consents. One monitoring visit at the investigator’s site prior to the start and twice (timing with the interim analyses) will be organized by the Sponsor. Furthermore, there will be a close-out visit at the study end.

#### Audits and inspections

A quality assurance audit/inspection of this study may be conducted by the competent authority. The quality assurance auditor/inspector will have access to all medical records, the investigator’s study-related files and correspondence, and the Informed Consent Form. The investigator will allow the persons being responsible for the audit or the inspection to have access to the source data/ documents and to answer any questions arising. All involved parties will keep the patient data strictly confidential.

### Participant timetable

For both SALATIO trials, we need 36 months each; starting in September 2022 (Table 2).

**Table 2:**
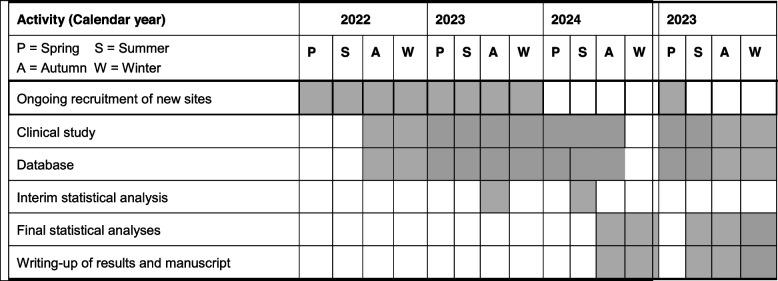
Time table of the study

The shaded periods correspond to the activity (items on the left) that will occur during the time schedule

### Outcomes of interest

Concerning the SALATIO trails, Table 3 summarizes the scheduled outcome parameters.

**Table 3:** Outcome parameters and assessments of both randomized trials

**Outcome(s)**	*Primary objectives:* • Clinical remission related to the duration of total, post-debridement, antibiotic use • Microbiological recurrence in relation to the total, post-debridement, antibiotic use *Secondary objectives*: - Description of all clinical failures of any sort - Adverse events in each study arm, and in relation to the antibiotics used - Length of hospital stay in acute care surgery (without rehabilitation)

Assessment of primary outcomes: Remission and microbiological recurrences of treated orthopedic infection at 12 months — Judged by local wound healing (healed vs delayed wound healing according to Reference [4]). Remission is defined as the absence of clinical, anamnestic, radiologic, or laboratory signs of former infection.

Assessment of other outcomes of interest: Secondary objectives are adverse events and length of hospital stay. We will note them during the study

### Statistical analyses and sample size

#### Main hypotheses

A 6 week’s course of targeted systemic antibiotic therapy after the first debridement for an orthopedic infection is not inferior to 12 weeks (if there are implants left in place). Likewise, a 3 week’s course of targeted systemic antibiotic therapy after the first debridement for orthopedic infection is not inferior to 6 weeks (if there are no implants).

We anticipate the corresponding short durations of post-debridement antibiotics reveal a similar remission incidence than the long ones. Provided that there is an adequate surgical strategy, the duration of antibiotic therapy does not modify remission nor microbiological recurrences, and antibiotic-related adverse events will occur in 10% of all treatment arms.

#### Sample size calculations

Both RCTs are non-inferiority trials. Remissions (at the first therapeutic approach) are set at 94% (6% recurrences in both arms). The maximum acceptable difference (unidirectional lower margin with binary-outcome categorical variables) is arbitrarily fixed at 10% regarding the primary outcome “remission.” Assuming a risk of alpha at 0.05 and a power of 80%, it will be necessary to recruit 70 patients in each antibiotic duration arm (short or long). Together with the distinction of the RCT into implant-related and implant-free bone infections, we would finally need 2 × 2 × 70 episodes, equaling a total of 280 episodes. With only very few anticipated dropouts, we would probably need 300 episodes. Our hospital approximately treats 600 episodes of different community-acquired and nosocomial infection episodes per year, of which roughly 150 are orthopedic infections. According to conservative estimates, we will see approximately 100 infection episodes per year that are eligible for the SALATIO trials. The interventional part of the trials will last 3 years (300 different episodes).

#### Planned analyses

All analyses will be performed for the entire study population and stratified upon the presence of an infected implant. In the second step, we will stratify the analyses basing on the orthopedic specialty. We will use descriptive statistics and perform group comparisons (using the Pearson-*χ*^2^-test, the Fisher exact, or the Wilcoxon-rank sum tests, as appropriate. We will also perform separate multivariate analysis using a Cox regression model [23]. Variables with a *p* value ≤0.2 in the univariate analysis will be included in a stepwise forward selection process for multivariate analysis. Key variables will be checked for co-linearity and interaction. The number of variables in the final model is limited to the ratio of 1 variable to 5 to 8 outcome events [23]. If the SALATIO trials expand to a multicenter study, or if there are more than 10% of the patients participating several times, we will add a cluster-specific analysis.

The intent-to-treat (ITT) population will consist of all randomized patients. The per-protocol (PP) population will consist of all randomized patients who complete the study without important deviations from the protocol.

#### Handling of missing data and drop-outs

Important missing data will lead to patient dropout of the study. Drop-outs will be reported in the Methods section of the publication. Drop-out data will be archived for a minimum of 10 years after study termination or in case of premature termination of the clinical trial.

#### Stratified analyses and multiple outcomes

The SALATIO trials will possess very complete data and perform stratified analyses (e.g., different entities of orthopedic infections and surgeries). Formal adjustments for multiple primary outcomes will not be a major problem. Principally, we have only two primary outcomes (clinical failures and microbiological recurrences), while the “microbiological recurrences” are part of the larger group of “clinical failures.” For this adjustment regarding both outcomes, we will formally use the “classical” Bonferroni correction that we consider as the most common approach to account for multiplicity due to its simplicity [24, 25].

#### Non-inferiority assessments

For assessment of the formal non-inferiority requirement (regarding the primary outcome “remission”), we will compute with a unidirectional *p*-value limit of 0.025. Formal non-inferiority assessment for “microbiological recurrence” will not be necessary, because of the few number of events anticipated. Likewise, we do not predefine a non-inferiority margin for secondary outcomes adverse events, or the length of hospital stay in acute care settings.

#### Interim analyses and early termination

We will perform two interim analyses, after 1 and 2 years, following the inclusion of the first patient. If the differences in the primary outcomes between the short and long antibiotic courses are important, or statistically significant [26], the independent Data Monitoring committee will decide upon the interruption or termination of the study (or, alternatively, to continue the trials in only one stratum). Otherwise, the study continues until the next interim analysis. The Data Monitoring committee has the right to call on a premature, additional interim analysis. If the group comparison analysis is not sufficiently meaningful, we will perform a formal futility analysis to check if the expected statistical power for the final analysis will not be unacceptable (more than 30%) [26]. If it is lower than 30%, we will consider the trial will not be able to demonstrate the result, and the recruitment will be no more ethical. The most frequent conditional power evaluated under the current trend (i.e., using the information from the collected data) will be assessed. Of note, it was demonstrated that futility analyses decrease the statistical power of the final analysis in superiority trials, but to our knowledge, this topic was not explored for non-inferiority trials [26]. To balance (at least partially) this loss of power, it is planned to recruit 50 supplementary patients per arm.

### Ethical and regulatory aspects

#### Study registration

The study is registered in the Swiss Federal Complementary Database (BASEC 2022-01012) and in the international registry ClinicalTrials.gov (Number NCT05499481).

#### Categorization of this study, safety reports, and eventual protocol modifications.

This study only makes use of antibiotics that are already authorized in Switzerland for orthopedic infections. The indication and the dosage are used in accordance with the prescribing information and international guidelines. The study protocol will not be changed without prior Sponsor’s and Ethical Committee’s approval. Premature interruption is reported within 30 days. As with the other RCTs we are currently conducting, and of which the protocols have been published in *Trials* [7, 10], we do not foresee to modify the protocol. If, however, there should be a protocol modification for any reason, we will ask the Ethical Committee by mail for the permission to amendments and/or modifications. The regular end of the study is reported to the Ethical Committee within 90 days. The final study report sis submitted within 1 year. The Ethical Committee and authorities will receive annual safety reports and are informed about the study’s end. The study will be carried out in accordance to the protocol and with principles enunciated in the Declaration of Helsinki, the guidelines of Good Clinical Practice (GCP), and the Swiss regulatory authority’s requirements.

#### Patient information and informed consent

Our institution has a standardized procedure for recruiting participants, based on the in- and exclusion criteria (Fig. 1). The investigators will inform potential participants about the study, its voluntary nature, procedures involved, expected duration, potential risks and benefits, and any potential discomfort. All participants will be provided with an information sheet and an informed consent form. The original form stays in the study records. For eventual BioBanking, the participants sign a General Consent regarding data and biologic material.

#### Participant privacy and confidentiality

The investigators uphold the principle of the participant’s right to privacy and that they shall comply with applicable privacy laws. Subject confidentiality will be further ensured by code numbers corresponding to the computer files. For verification, the Ethics Committee and regulatory authorities may require access to medical records, including the medical history.

#### Early termination of the study

The Sponsor may terminate the study prematurely in certain circumstances, e.g., ethical concerns, insufficient recruitment, when the safety of the participants is at risk, respectively, alterations in accepted clinical practice making the continuation unwise, and early evidence of benefit or harm of the experimental intervention. All patients are free to withdraw from participation in this study at any time, for any reason, and without prejudice. The reason for withdrawal should be documented wherever possible. The withdrawal will not affect the actual medical assistance or future treatments. On rare occasions, the investigators may terminate a patient’s participation to protect his/her best interest. After study termination, the evaluations required at the next scheduled clinical visits will remain.

### Safety

All surgeries will be performed in the participation of an experienced surgeon. The antibiotic therapy is ordered and supervised by internists and infectious diseases physicians with experience in orthopedic infections.

#### Definition and assessment of (serious) adverse events and other safety-related events

An Adverse Event (AE) is any medical occurrence in a study participant, which does not necessarily have a causal relationship with the study procedure. An AE can therefore be any unfavorable and unintended symptom. A Serious Adverse Event (SAE) is classified as any untoward medical occurrence that results in death, is life-threatening, requires in-patient hospitalization or a significant prolongation of hospitalization, persistent or significant disability. Participants with ongoing SAEs at study termination will be followed until recovery or stabilization after termination. The investigators make a causality assessment. All SAEs are reported within 24 h to the Sponsor-Investigator. SAEs resulting in death are reported to the Ethics Committee within 7 days. The Sponsor-Investigator will report the safety signals within 7 days to the local Ethics Committee. Patients who will AE and leave the study will be treated off-study, without restriction, at the study sites.

#### Periodic reporting of safety

An annual safety report on the participant is submitted once a year to the local Ethics Committee. Moreover, we perform annual interim (futility) analyses.

#### Data handling and record keeping/archiving

Data is only saved using the secured software REDCap®. When the study is terminated, it will be saved in the same system. Data can only be accessed by defined persons that are investigators of the project.

#### Case report forms

An electronic Case Report Form will be generated for every participant and all data relevant to the study is going to be recorded by authorized persons. The participant ID numbers are automatically assigned in consecutive ascending form by the REDCap® system. Corrections can only be made by authorized persons.

#### Analysis and archiving

For data analysis, subject-related data from REDCap will be exported and analyzed in a statistical software (IBM-SPSS and/or STATA). All health-related data will be archived in the REDCap®. Before data export, all patient identifiers are removed. All data will be stored for a minimum of 10 years. Collection, disclosure, and storage of data are carried out in accordance with Swiss data protection regulations and the Human Research Act. The BioBanking stores the eventual intraoperative samples in accordance with laboratory guidelines as standard.

## Discussion

The SALATIO trials shall demonstrate a clinically relevant non-inferiority (10% margin) “in favor” of a significantly shorter antibiotic therapy, compared to the usual long durations in adult patients with various orthopedic infections. This reduction shall concern infections with and without implants [27]; independently of the surgical technique, the pathogens, the number of debridements, the infection localization, or the presence of immune suppression. Maybe we will also witness less AE in the shorter randomization arms. Ultimately, we want to reduce the post-surgical antibiotic duration to half of what would prescribe by the majority of clinicians; at least for “usual” (standard) infection cases.

In the SALATIO trials, we principally keep a longer antibiotic duration for implant infections, even if we ignore if implant-related infections really must be treated longer. Basically, there are no firm clinical data suggesting that the prolongation of antibiotic prescription should exactly be doubled for implants compared to implant-free bone infections. In the theory and according to in vitro studies, the foreign body nature of the implant makes every infection difficult to treat, even for pathogens not usually considered as virulent [2], Unsurprisingly, many experts group recommend antibiotic therapy for 3 months for hip arthroplasties (sometimes up to 6 months for total knee arthroplasties) with arthroplasty retention and 6 weeks for two-stage exchanges. This is clearly exaggerated. Nevertheless, in the set-up of the SALATIO trials, we keep the philosophy for foreign material infections, to comply with the traditions.

In contrast, our approach is different from the tradition for implant-free bone infections. In the entire field of orthopedic bone infections, there are very few and inconsistent scientific data proving the benefit of a systemic antibiotic therapy beyond 6 weeks; compared to 4 to 6 weeks or even less. Exceptions are by nature expert opinions in book chapters or past publications without own database analyses; or the therapy of special microorganisms requiring long-lasting antibiotic therapies such as mycobacteria [28], *Nocardia* spp. [29], actinomyces or fungi [10]. To cite our own examples in bone-related infections, sacral osteomyelitis [16], long bone osteomyelitis [9], fracture-device-related infections [8, 30], prosthetic joint infections [1, 4], diabetic foot osteomyelitis (DFO) [3] and many more failed to enhance remission rates, if antibiotics were prolonged beyond 4 to 6 weeks. For instance, Bernard et al. performed a multicenter prospective trial randomizing vertebral osteomyelitis between 6 and 12 weeks and showed similar results in both study arms [2]. Tone et al. randomized non-amputated DFOs between 6 and 12 weeks and found no differences in remission rates [31]. Chaussade et al. retrospectively analyzed the outcome of arthroplasties treated with debridement and retention and found that, despite implant retention, a 6 weeks course of antibiotics was not inferior to 12 weeks [1]. Farhad et al. resumed that 6 weeks of antibiotic therapy was sufficient for all bone and implant infections [5]; together with an early switch to oral medication [32]. These relatively short durations are equally acknowledged by international consensus meetings [33] of orthopedic surgeons and infectious diseases experts, who treat these infections.

There are also studies with less than 6 weeks of total antimicrobial therapy, especially in the pediatric literature for hematogenous osteomyelitis and/or septic arthritis [34, 35]. In this particular setting, a 3-week”s course appears to be sufficient [36–38]. To cite some examples among adult patients, the results of our single-center, real-life, pilot study in adult patients with DFO patients found that a relatively short course of post-debridement antibiotic prescription (3 weeks) was non-inferior to the current standard duration of 6 weeks [3]. Clinical remission at 2 months was at 78%, which is similar to that reported in other published DFO series [3]. We also advocated for only 4 weeks in operated implant-free osteomyelitis. Our randomized trial involving 123 adult patients found no significant difference in outcomes in patients treat with 4, compared to 6, weeks of antibiotic therapy after removal of the implant [8]. Similarly, there was no significant difference between the groups in the rate of microbiological cures, antibiotic-related AE, or any other outcomes [8].

In our era of critical shortages of effective antibiotics, a shorter treatment duration could decrease antibiotic-related adverse events, costs, and possibly the emergence of resistance. We do not expect major difficulties performing in the SALATIO trials, provided that patients and surgeons agree to participate. Despite two prospective-randomized designs (for infections with and without implants), and only 280 different episodes anticipated, the patients’ voluntary participation might be low. Likewise, patients who are continued to be treated outside of our hospital may have been lost, or have their treatment changed, because the following clinicians might not agree or may lack interest. However, our center is the largest public hospital for orthopedic infections in the region; so, this is unlikely to be a major bias. Lastly, the SALATIO trials exclude episodes with a strictly conservative (non-surgical) treatment of orthopedic infections or management using systemic antibiotics in conjunction with local, intraosseous antibiotic deliveries [19]. Hence, our results should not be confounded with a conservative, non-surgical approach to these infections. Similarly, the requirement of a professional debridement, curettage, or implant removal by experienced orthopedic surgeons is not available everywhere and represents a “hospital bias” regarding the results. A multicenter approach might circumvent this bias, but also reduce the pooled success [15]. Nevertheless, according to our protocol, the SALATIO trials is open for a multi-site participation of experienced centers.

In conclusion, we are confident to finish the SALATIO trials, reveal some answers to frequent questions regarding the post-surgical duration of systemic antibiotic use in orthopedic infections, and avoid unnecessary antibiotic excesses, all of which we consider as a personal mission and commitment [39].

## Supplementary Information

**Additional file 1: Supplementary File 1.** Protocol SALATIO Trials.

**Additional file 2: Supplementary File 2.** Consent Form in English language. SALATIO Trials.

**Additional file 3: Supplementary File 3.** Internal Document and Proof of Funding.

## Data Availability

We may provide anonymized key elements of the datasets upon reasonable scientific request.
