# Supplementary material for: Study protocol: short against long antibiotic therapy for infected orthopedic sites — the randomized-controlled SALATIO trials
Source: Trials. 2023 Feb 18;24:117. doi: 10.1186/s13063-023-07141-2 (PMC9938993; doi:10.1186/s13063-023-07141-2)
Supplement: Supplementary file 1 — Additional file 1: Supplementary File 1. Protocol SALATIO Trials. [file 13063_2023_7141_MOESM1_ESM.pdf]

Clinical Study Protocol

Short Against Long Antibiotic Therapy for Infected Orthopedic Sites - SALATIO trials

Study Type:	Clinical trial with investigational drug
Study Categorisation:	Risk category A
Study Registration:	Clinicaltrials.gov und SNCTP
Sponsor-Investigator:	Prof. Dr. med. Mazda Farshad<br>Medical Director, surgeon-in-Chief, Orthopaedic Department<br>Balgrist University Hospital<br>Forchstrasse 340<br>8008 Zürich<br>044 386 1111<br>ilker.uckay@balgrist.ch
Principal Investigator:	Prof. Dr. med. Ilker Uçkay;<br>Head Infectiology, Head Clinical Research in Orthopaedic Department<br>Balgrist University Hospital<br>Forchstrasse 340<br>8008 Zürich<br>044 386 1111<br>ilker.uckay@balgrist.ch
Investigational Products:	None; antibiotics freely available on the Swiss market
Protocol Version and Date:	Version 1.0, 19.05.2022

CONFIDENTIAL

The information contained in this document is confidential and the property of the department of orthopaedics and infectiology of the Balgrist University Hospital. The information may not - in full or in part - be transmitted, reproduced, published, or disclosed to others than the applicable Competent Ethics Committee(s) and Regulatory Authority(ies) without prior written authorisation from Balgrist University Hospital, except to the extent necessary to obtain informed consent from those who will participate in the study.

SIGNATURE PAGE

Study Title **Short Against Long Antibiotic Therapy for Infected Orthopedic Sites - SALATIO trials**

The Sponsor-Investigator and trial statistician have approved the protocol version 1.0, 19.05.2022, and confirm hereby to conduct the study according to the protocol, current version of the World Medical Association Declaration of Helsinki, ICH-GCP guidelines or ISO 14155 norm if applicable and the local legally applicable requirements.

Sponsor-Investigators: Prof. Dr. med. Mazda Farshad0

Zürich, 19.05.2022

Place/Date Signatures

Principal Investigators:

I have read and understood this trial protocol and agree to conduct the trial as set out in this study protocol, the current version of the World Medical Association Declaration of Helsinki, ICH-GCP guidelines or ISO 14155 norm and the local legally applicable requirements.

Site: Balgrist University Hospital
Forchstrasse 340
8008 Zürich
Switzerland

Principal Investigators: Prof. Dr. med. Ilker Uçkay

Zürich, 19.05.2022

Place/Date Signatures

TABLE OF CONTENTS

SIGNATURE PAGE ... 2
TABLE OF CONTENTS ... 3
STUDY SUMMARY IN LOCAL LANGUAGE ... 9
ABBREVIATIONS ... 10
STUDY SCHEDULE ... 11
1. STUDY ADMINISTRATIVE STRUCTURE ... 12
1.1 Sponsor-Investigator ... 12
1.3 Statistician ("Biostatistician") ... 12
1.4 Laboratory ... 12
1.5 Monitoring institution ... 12
1.6 Data Safety Monitoring Committee ... 12
1.7 Any other relevant Committee, Person, Organisation, Institution ... 12
2. ETHICAL AND REGULATORY ASPECTS ... 12
2.1 Study registration ... 12
2.2 Categorization of study ... 12
2.3 Competent Ethics Committee (CEC) ... 12
2.4 Competent Authorities (CA) ... 13
2.5 Ethical Conduct of the Study ... 13
2.6 Declaration of interest ... 13
2.7 Patient Information and Informed Consent ... 13
2.8 Participant privacy and confidentiality ... 13
2.9 Early termination of the study ... 14
2.10 Protocol amendments ... 14
3. BACKGROUND AND RATIONALE ... 14
3.1 Background Rationale ... 14
3.2.1 Standard antibiotic prophylaxis used in the study ... 15
3.2.2 Definitions of an orthopedic infection ... 15
3.3 Clinical Evidence to Date ... 15
3.4 Dose Rationale ... 15
3.5 Explanation for choice of comparator ... 15
3.6 Risks/Benefits of the study and of BioBanking ... 16
3.7 Justification of choice of study population ... 16
4. STUDY OBJECTIVES ... 16
5. STUDY OUTCOMES ... 16
6. STUDY DESIGN ... 16
6.1 General study design and justification of design ... 16
6.1.3 Study duration ... 17
6.2 Methods of minimizing bias ... 17
6.2.1 Method of assignment to treatment/intervention (randomization, stratification) ... 17
6.2.2 Blinding procedures ... 17
6.3 Unblinding Procedures ... 17
7. STUDY POPULATION ... 18
7.1 Eligibility criteria ... 18
7.2 Recruitment and screening ... 18

7.3 Assignment to study groups 18
7.4 Criteria for withdrawal / discontinuation of participants 18
8. STUDY INTERVENTION 19
8.1 Investigational Products (treatment / medical device) 19
8.1.1 Intervention treatment 19
8.1.2 Control Comparator....19
8.1.3 Packaging, Labelling and Supply (re-supply) 19
8.1.4 Storage Conditions....19
8.2 Administration of experimental and control interventions 19
8.2.1 Experimental Intervention 19
8.2.2 Control Intervention....19
8.3 Dose modifications 19
8.4 Compliance with study intervention....19
8.5 Data Collection and Follow-up for withdrawn participants 19
8.6 Trial specific preventive measures 19
8.7 Concomitant Interventions (treatments) 20
8.8 Study Drug Accountability 20
8.9 Return or Destruction of Study Drug 20
9. STUDY ASSESSMENTS 20
9.1 Assessment of primary outcome....20
9.1.2 Assessment of other outcomes of interest....20
9.1.3 Assessment of safety outcomes 20
9.1.4 Assessments in participants who prematurely stop the study 20
9.2 Procedures at each visit for both prospective-randomized studies 20
9.2.1 Screening/Pre-procedure assessment/Visit 1....21
9.2.2 Visit 2, 3, 4, and End of Treatment 21
9.2.3 Visit 5 (Test of Cure) 21
9.2.4 Early Termination of Study Patients....21
10. SAFETY 21
10.1 Drug studies 21
10.1.1 Treatment by specialists at UKB....21
10.1.2 Definition and assessment of (serious) adverse events and other safety related events ...21
10.1.3 Reporting of serious adverse events (SAE) and other safety related events 22
10.1.4 Follow up of (Serious) Adverse Events....23
11. STATISTICAL METHODS.... 23
11.1 Main hypotheses 23
11.2 Determination of Sample Size....23
11.3 Planned Analyses....23
11.3.1 Interim analyses and early termination 24
11.3.2 Final analyses 24
11.4 Handling of missing data and drop-outs....24
12. QUALITY ASSURANCE AND CONTROL.... 24
12.1 Data handling and record keeping / archiving....24
12.1.1 Case Report Forms....25
12.1.2 Specification of source documents 25
12.1.3 Record keeping / archiving 25

12.1.4 BioBanking ... 25
12.2 Data Management System, access and back-up ... 25
12.2.1 Analysis and archiving ... 25
12.3 Monitoring ... 26
12.4 Audits and Inspections ... 26
12.5 Confidentiality, Data Protection ... 26
12.6 Storage of biological material and related health data ... 26
13. PUBLICATION AND DISSEMINATION POLICY ... 26
14. FUNDING AND SUPPORT ... 27
15. INSURANCE ... 27
16. REFERENCES ... 28

STUDY SYNOPSIS

**Sponsor-Investigator(s)**	Prof. Dr. med. Mazda Farshad, Medical Director
**Principal Investiga-tors**	Prof. Dr. med Ilker Uçkay, Head of Infectiology, Head of the Unit for Clinical and Applied Research;
**Study Title:**	**S**hort **A**gainst **L**ong **A**ntibiotic **T**herapy for **I**nfected **O**rthopedic Sites<br>- **SALATIO** trials
**Short Title / Study ID:**	The **SALATIO** trials
**Protocol Version and Date:**	Version 1.0; 19.05.2022
**Trial registration:**	Swiss National Clinical Trials Portal (SNCTP) and the international trial registry ClinicalTrials.gov (www.clinicaltrials.gov). Publication of the protocol in the scientific journal "*Trials*".
**Study category and Rationale**	Category A: All antibiotics, with indications, used are authorized by *Swissmedic* and are used standardly in clinical practice
**Clinical Phase:**	Not applicable; the prophylactic antibiotics are used according to the prescribing information
**Background and Rationale:**	The optimal duration of postoperative, systemic antibiotic therapy for implant-related orthopedic infections, with or without implant removal, is unknown[1-12].<br>Retrospective studies suggest that a maximum duration of 6 weeks is not inferior to longer administrations[1-5]; even if the infected implants are kept in place[1,3] or during a one-stage exchange[2]. Prospective-randomized trials (RCT) suggest that even shorter durations, such 3 or 4 weeks[6-10], are possible, when the implant is removed. Likewise, in prospective studies, 6 or 8 weeks of systemic antibiotics are not inferior[10-12] to the current 12 weeks during DAIR (debridement, antibiotic and implant retention)[1,3,12], or during the one-stage exchange[2,4]; except for one single RCT suggesting a better outcome for 12 weeks in the substrata of arthroplasty infections undergoing the DAIR procedure[13]. However, these RCTs concern selected branches of orthopedic surgery; especially prosthetic joint infections. We intend to expand these evaluations (with new RCTs) to all fields of orthopedic and hand surgery. The only exceptions would be spine surgery, for which a multicenter, separate RCT is already under way (SASI-trials)[10]. The second exception would be the treatment of implant-free diabetic foot infections, for which two distinct RCTs are already under way[7].

**Study Conduct**	We will perform two concomitant RCTs, depending on the presence of infected osteosynthesis material at enrolment:<br>- SALATIO 1. Infected implant not removed (or new material inserted):<br>Randomization 6 vs. 12 weeks (+/- 5 days) of total antibiotic therapy counted since the first debridement for infection. Early switch to oral targeted therapy.<br>- SALATIO 2. Infected implant without residual material (definitive removal or within the interval of a two-stage exchange):<br>Randomization 3 vs. 6 weeks (+/- 5 days) of total antibiotic therapy counted since the first debridement for infection. Early switch to oral targeted therapy.<br><br>After randomization, the study participants will be actively followed-up for a minimum of 12 months in case of retained implants or musculoskeletal grafts; or for 2 months without implants or grafts. At database closure, we will review the medical charts of all patients to seek for unscheduled visits since the inclusion. This "passive follow-up" can reach up to four years and terminates at the date of database closure. The scheduled study visits take place as follows:<br>Visit 1 - Enrollment (Day 1),<br>Visit 2 - Day 21 (+/- 5 days),<br>Visit 3 - Day 42 (+/- 5 days) (standard surgical orthopedic control in our setting)<br>Visit 4 - Day 84 (+/- 5 days).<br>End of treatment (EOT) visit - Day 21, 42 or Day 84 (+/- 5 days) (only if still receiving treatment after visit 2).<br>Test-of-cure (TOC) visits - 6 months (+/- 2 weeks)<br>Follow-up visit - 12 months (+/- 2 months) with implants; 6 months (+/- 2 months) without implants.<br><br>During the study visits, we assess the history, adverse events, and the functional status. We examine the patients according to the orthopedic standard, and add supplementary laboratory exams and radiology, only if clinically indicated. The visit 2 can be by telephone. If the patient is currently hospitalized for rehabilitation under medical supervision, the visits can be replaced by the medical assessment during the reeducation/rehabilitation.<br><br>**Antibiotic agents**<br>The antibiotic therapy is prescribed by Infectious Diseases physicians with experience in orthopaedic infections, the surgeons in charge of the patient, and/or the internists. The antibiotic therapy is either empiric or targeted to the results of at least three intraoperative, microbiological samples from bone or metal. The choice of the agent, or its administration route, are at the discretion of treating clinicians. However, for this study, and in order to achieve a minimal homogeneity, we established a list of "allowed antibiotics" and their recommended doses.
**Objective(s):**	To reduce the post-debridement antibiotic duration in all sorts of orthopedic in adult patients.<br>We evaluate if 6 weeks of systemic and targeted antibiotic therapy postoperatively is not inferior to 12 weeks (non-inferiority trial) in case of infections with a (new) implant (DAIR procedure or one-stage exchange), after a follow-up of 12 months. For orthopedic infections with complete implant removal (or with external fixation only), the objective is the evaluation if 3 weeks of antibiotic therapy is not inferior to 6 weeks.<br>Secondary objectives are the assessments of differences regarding adverse events
**Outcome(s)**	*<u>Primary objectives</u>:*<br>• Clinical remission related to the duration of total, postdebridement, antibiotic use<br>• Microbiological recurrence in relation to the total, postdebridement, antibiotic use<br>*<u>Secondary objectives</u>*:<br>- Description of all clinical failures of any sort<br>- Adverse events in each study arm, and in relation to the antibiotics used<br>- Length of hospital stay in acute care surgery (without rehabilitation)<br>- BioBanking of infected tissue for ultimate laboratory studies
**Study design**	Two concomitant, separate, prospective-randomized, non-inferiority trials

**Study criteria:**	Inclusion criteria	• Age ≥ 18 years<br>• Orthopedic bone and implant infections, including sacral osteomyelitis and musculoskeletal grafts<br>• Intraoperative debridement with any surgical technique<br>• 12 months of scheduled follow-up from hospitalization<br>• Bacterial orthopedic infections of any nature, independently of implants or co-morbidities; according to clinical, laboratory, radiological, microbiological features of infection<br>• First or second episode of infection
	Exclusion criteria	• Mycobacterial, fungal, nocardial, and *Actinomyces* infections<br>• Purely soft tissue infections<br>• Non-resected cancer in the infection site<br>• Purely intrasynovial infections (native joint septic arthritis)<br>• More than three debridements performed for infection<br>• Absence of at least one surgical intraoperative debridement<br>• Spine infections (investigated in another trial)[10]<br>• Diabetic foot infections (investigated in another trial)[7]<br>• Documented endocarditis according to the Duke criteria<br>• At least 2 prior infection episodes at the actual infection site<br>• Inability to understand the study procedure for language or cognitive reasons
**Definitions**	Implants are defined as any implants except for transient wires or fixator pins.<br>An infection is defined as having ≥ 2 local manifestations of inflammation (swelling or induration, erythema, local tenderness or pain, local warmth, purulent discharge); together with the same pathogen(s) retrieved in the microbiological culture of at least two intraoperative samples.<br>Remission is defined as the absence of any clinical, anamnestic, radiological or laboratory signs of former (or new) surgical site infections within a minimal follow-up of 12 months (longer follow-ups are permitted). A diagnostic control puncture for the microbiological exclusion of dormant bacteria is not necessary.	
**Limitation**	Heterogenous group of orthopedic infections. However, the literature makes no differences in terms of antibiotic therapy in all strata of implant infections.	
**Measurements and procedures.**	We will assess:<br>– Patient's characteristics: age, sex, known immune-suppression (diabetes mellitus, renal dialysis, cirrhosis, pregnancy, medicamentous immune-suppression, untreated HIV disease, agranulocytosis, active cancer), American Society of Anesthesiologists' (ASA)-Score<br>– Surgery specific baseline data: number and type of surgeries for the actual problem, agent, dose and duration of pre-surgical antibiotic therapy, local antibiotics used in the bone, cell count (if any), initial serum C-reactive protein (CRP) level, presence of initial bacteremia.<br>– Anatomical localization of surgery, type of surgery, microbiological results, histology (facultative)<br>– Treatment and outcome: number of surgeries to treat infection, total duration of antibiotic therapy, duration, agent and dose of intravenous and oral antibiotic therapy, wound healing problems, presence and duration of vacuum-assisted negative pressure therapy, adverse events, clinical or and microbiological recurrence, date and reasons for re-hospitalization and re-treatment, follow-up data, fatalities<br>– Administrative data: total hospitalization length, BioBanking of infected tissues at the Balgrist Campus	
**Expected results**	Three weeks of post-debridement antibiotics reveal the same overall remission in implant-removed orthopedic infections than six weeks.<br>Six weeks of post-debridement antibiotics reveal the same remission rate in implant-related orthopedic infections than twelve weeks.<br>The duration of antibiotic therapy does not modify clinical outcome nor microbiological recurrences<br>Antibiotic-related adverse events will occur in 10% of all treatment strata.	
**Impact**	Future patients with orthopedic infections will benefit from shorter antibiotic therapies, shorter hospital stays, thus reducing adverse events of antibiotic treatment. Finally, our project is a contribution to the professional efforts of antibiotic stewardship that is gaining momentum worldwide.	

**Study Product**	None. All antibiotics are freely available on the Swiss market.
**Statistical analyses**	Both RCT are non-inferiority trials. Remissions (at the first therapeutic approach) are set at 94% (6% recurrences in both arms). The maximum acceptable difference (unidirectional lower margin with binary-outcome categorical variables) is arbitrarily fixed at 10% regarding the primary outcome remission. Assuming a risk of alpha at 0.05 and a power of 80%, it will be necessary to recruit 70 patients in each antibiotic duration arm (short or long). Together with the distinction of the RCT into implant-related and implant-free bone infections, we would finally need 2 x 2 x 70 episodes, equaling a total of 280 episodes within three years; including for all strata of orthopedic infections. With the dropouts, we would probably need 300 episodes. For assessment the formal non-inferiority requirement (regarding the primary outcome “clinical remission”), we will compute with a unidirectional p-value limit of 0.025. We do not predefine a non-inferiority margin for secondary outcomes such microbiological recurrences, adverse events or functional outcomes.<br><br>*Interim analyses*<br>We perform two interim analyses after approximatively one and two years. On this occasion, we check if the expected statistical power for the final analysis will be acceptable. If it is lower than 30%, we will consider the trial will not be able to demonstrate the result, and the recruitment is no more ethical. A Study Monitoring Committee will consist of independent clinicians, with statistical experience, and not participating in the study. They will decide about the future of the trials.<br><br>The intent-to-treat (ITT) population will consist of all randomized patients who signed for the participation. The per-protocol (PP) population will consist of all patients who complete the study and who have not deviated significantly from the protocol. The statistical analyses will mostly base on descriptive analyses, group comparisons and a multivariate, unmatched, eventually cluster-controlled, Cox regression analysis adjusting for the large case-mix that we expect.
**Number of Participants with Rationale:**	The Balgrist University Hospital treats approximately 600 episodes of different community-acquired and nosocomial infections per year, of which roughly 150 orthopedic infections. According to our conservative estimates, we will see approximatively 100 infection episodes per year that are eligible for the SALARIOM trials. We expect the trials finished within three years (280 patients). The study starts at the Balgrist, but can be expanded to other sites with active orthopedic research.
**GCP Statement:**	This study will be conducted in compliance with the protocol, the Declaration of Helsinki, the ICH-GCP and national legal and regulatory requirements.

STUDY SUMMARY IN LOCAL LANGUAGE

Die optimale Dauer der systemischen, antibiotischen Begleittherapie nach erfolgtem chirurgischem Debridement (oder Entfernung des infizierten Implantates) ist unbekannt.

Bei liegenden infizierten orthopädischen Implantaten wird in der Schweiz und in andren Ländern meist während 12 Wochen Antibiotika verabreicht, in anderen nur für 6 Wochen. Im Balgrist führen wir bereits seit zwei Jahren mehrere prospektive-randomisierte Trials durch, wobei wir die entsprechenden Antibiotikadauern zwischen 6 und 12 Wochen (Wirbelsäule) oder 3 bis 6 Wochen (diabetische Fuß-Osteomyelitis) festlegen. Beide RCT, die als "Vorspur" zur aktuellen Studie zeigen in den Interimsanalysen keine Unterschiede bezüglich Infektheilung zwischen dem kurzen und den langen Antibiotikaarm. Diese Balgrist-Daten wurden vorher bestätigt durch zwei andere RCT, welche Mitglieder unseres Teams in Genf durchführen konnten: 3 versus 6 Wochen in diabetischer Fuß-osteomyelitis und 4 versus 6 Wochen Antibiotikatherapie für Implantat-Infektionen mit Entfernung des Implantats; und vieler anderer retrospektiver Studien. Diese Studien sind klinisch relevant, da sie sich dem Zeitgeist einer optimierten Antibiotikatherapien widmen.

In dieser sehr ähnlichen, neuen Studie erforschen wir die Antibiotikadauer bei anderen orthopädischen Infektionen (Fuß-Infektionen mit belassenem Implantat; Schulter, Knie, Hand und Hüft-Infektionen sowie operierte sakrale / trochantäre Osteomyelitis-Falle mit oder ohne Lappendeckung). Wir randomisieren wiederum zwischen 6 und 12 Wochen antibiotischer Therapie bei liegendem, infiziertem Implantat, und zwischen 3 und 6 Wochen, wenn dieses vollständig entfernt wurde. Bedingung ist, dass alle Patienten mindestens ein gründliches, chirurgisches Debridement im Operationssaal hatten.

Diese jetzige Studie ist im Prinzip die gleiche prospektiv-randomisierte Studie, welche aber die bisher vernachlässigten Disziplinen der orthopädischen Studie miteinschließt. Die nötige Studienerfahrung in dieser Fragestellung haben wir schon. Die angedeuteten Studien in der Wirbelsäulen-Chirurgie und dem diabetischen Fuß laufen separat weiter.

ABBREVIATIONS

AE	Adverse Event
ASA	American Society of Anesthesiologists
ASR	Annual Safety Report
CA	Competent authority
CEC	Competent Ethics Committee
CRP	Serum C-reactive protein
eCRF	Electronic case report forms
EOT	End of treatment
GCP	Good Clinical Practice
ICH-GCP	International Conference on Harmonization of Good Clinical Practice
IMM	Institut für Medizinische Mikrobiologie
NRS	Nutritional Risk Screening
PD	Privatdozent
REDCap	Research Electronic Data Capture
SAE	Serious Adverse Events
SNCTP	Swiss National Clinical Trials Portal
SOP	Standard Operation Procedure
SUSARs	Suspected Unexpected Serious Adverse Reactions
UCAR	Unit for Clinical and Applied Research
UKB	Universitätsklinik Balgrist
VAC	vacuum-assisted negative pressure
ZLZ	Zentrallabor Zürich

STUDY SCHEDULE

Randomized-controlled trials

Study Periods	Screening/ Baseline*	Visit 1*	Visit 2*	Visit 3* End-of-Treat-ment	Visit 4* End-of-Treatment	Test-of-cure
**Time**	-30 to 0	21 (+/- 5d)	42 (+/- 5 d)	21 or 42 (+/- 5d)	42 or 84 (+/- 5d)	6 or 12 mts. (+/- 2 months)
In-/ Exclusion criteria	X					
Informed consent	X					
Demographics / history[1]	X					
Randomization	X					
Concomitant medication		X	X	X	X	
Compliance		X	X	X	X	
Adverse Events		X	X	X	X	X
Study End[2]						X
BioBanking	X					

[1] Patient's characteristics: age, sex, known immune-suppression (diabetes mellitus, renal dialysis, cirrhosis, pregnancy, medicamentous immune-suppression, untreated HIV disease, agranulocytosis, active cancer), American Society of Anesthesiologists' (ASA)-Score

Surgery specific baseline data: number and type of surgeries for the actual problem, agent, dose and duration of pre-surgical antibiotic therapy, local antibiotics used in the bone, cell count (if any), initial serum CRP level, presence of initial bacteremia.

Anatomical localization of surgery, type of surgery, microbiological results, histology (facultative)

[2]Treatment and outcome: number of surgeries to treat infection, total duration of antibiotic therapy, duration, agent and dose of intravenous and oral antibiotic therapy, wound healing problems, presence and duration of vacuum-assisted negative pressure therapy, adverse events, clinical or and microbiological recurrence, date and reasons for re-hospitalization and re-treatment, follow-up data, fatalities

[2]Administrative data: total hospitalization length, BioBanking of infected tissues at the Balgrist Campus

*The visits are all standard. The data is collected from the medical record.

1. STUDY ADMINISTRATIVE STRUCTURE

1.1 Sponsor-Investigator

Prof Dr. med. Mazda Farshad, Medical Director, Balgrist University Hospital, Forchstrasse 340, 8008 Zürich

The sponsor is responsible for trial design and management, data handling and record keeping, subject protection, quality management, financing, investigational product management and safety evaluation. He ensures oversight and designates appropriately qualified personnel. The sponsor is going to supervise data collection, management and integrity as well as analysis and interpretation.

1.2 Principal Investigator:

Prof Dr. med. Ilker Uçkay, Head Infectiology, Head UCAR (Clinical Research in Orthopedic Department).

The PI is responsible for the protocol and GCP conform conduct of the trial at the site. She delegates and supervises trial-related duties to qualified staff and ensures medical care of trial subjects. The PI ensures that randomization and informed consent procedures are followed, source and CRF records are accurate and that safety reporting requirements are met.

1.3 Statistician ("Biostatistician")

Statistical analyses will be performed by the investigators (and eventually the biostatistician Mr. Tobias Götschi of UCAR (Unit for Clinical and Applied Research) using SPSS™ and/or STATA™ software (Version 14). In case of necessity, other biostatisticians will be consulted.

1.4 Laboratory

Laboratory analysis will be done by ZLZ Zentrallabor Zürich and IMM, in the immediate vicinity of UKB, as part of the regular analysis of the clinical course.

Intraoperative samples of infected tissues might be asserted and stored at the Biobank Balgrist Campus.

1.5 Monitoring institution

An internal study monitoring board is established to perform ongoing study surveillance and to perform interim analyses if appropriate. UCAR (Unit for Clinical and Applied Research); Prof Dr. med. Ilker Uçkay, Balgrist Campus, Lengghalde 5, 8008 Zürich

1.6 Data Safety Monitoring Committee

A data safety committee of two persons with experience in clinical research and biostatistics who are not part of the co-investigators or future authors of the scientific publication will monitor the safety of data and of the study; during the two interim analyses (approximatively after one and two years into the study).

1.7 Any other relevant Committee, Person, Organisation, Institution

The study starts at the Balgrist University Hospital, but can be expanded to other centres in Switzerland and abroad (with amendments to the Ethical Committees)

2. ETHICAL AND REGULATORY ASPECTS

The decision of the Competent Ethics Committee (CEC) concerning the conduct of the study will be made in writing to the Sponsor-Investigator before commencement of this study. The clinical study can only begin once approval from the CEC has been received. Any requirements imposed by the authorities shall be implemented.

2.1 Study registration

The study will be registered at http://www.clinicaltrials.gov and http://www.snctp.ch.

2.2 Categorization of study

Category A. This study only makes use of the medicinal products which are already authorized in Switzerland for the treatment of surgical sites infections, including with materials. The indication and the dosage are used in accordance with the prescribing information and the international guidelines. All drugs and doses in this study are commonly used agents and freely available on the Swiss market since decades. There will be no placebo.

2.3 Competent Ethics Committee (CEC)

The principal investigator ensures that approval from an appropriately constituted Competent Ethics Committee

(CEC) is sought for this clinical study.

The reporting duties such as all changes in research activity, all unanticipated problems involving risks to humans and planned or premature study end and the allowed time frame are respected by this study. The study protocol will not be changed without prior Sponsor and CEC approval, except when it's necessary to eliminate apparent immediate hazards to study participants.

Premature study end or interruption of the study is reported within 15 days. The regular end of the study is reported to the CEC within 90 days, the final study report shall be submitted within one year after study end. Amendments are reported according to chapter 2.10.

2.4 Competent Authorities (CA)

CA (*swissmedic*) approval is only necessary for category B and C studies. Category A studies do not require CA approval. The CA is entitled to carry out inspections of all clinical trials.

2.5 Ethical Conduct of the Study

The study will be carried out in accordance to the protocol and with principles enunciated in the current version of the Declaration of Helsinki, the guidelines of Good Clinical Practice (GCP) issued by ICH, the Swiss Law and Swiss regulatory authority's requirements. The CEC and regulatory authorities will receive annual safety and interim reports and be informed about study stop/end in agreement with local requirements.

2.6 Declaration of interest

No conflict of interest compromises the professional judgement of our investigators and the other involved people in conducting and reviewing this study. Their objectivity is not influenced in any way (e.g. independence, intellectual, financial, proprietary) or through any party.

2.7 Patient Information and Informed Consent

For the participation in the study, patients will be recruited/preselected by any of the investigators of the study. If patients match the inclusion criteria and do not meet any exclusion criterion for study, they will be informed by one of the study investigators, about the study, its nature, purpose, procedures involved, expected duration, participating investigators, potential risks and benefits and any potential discomfort the study could entail, during post-surgery visit when an orthopedic infection is diagnosed. Each participant will be informed that the participation in the study is completely voluntary and that he/she may withdraw from the study at any time and that withdrawal of consent will not affect his/her medical assistance and treatment in the future. No further screening requirements (other than the in- and exclusion criteria) exist.

All participants will be provided a participant information sheet and informed consent form describing the study and entailing sufficient information for the participant to make an informed decision about their willingness to participate in the study. The patient information sheet and the consent form will be submitted to the CEC to be reviewed and approved. The information sheet provides the possibility to read through the study concept again and enables the patient to rethink the study participation without being pressured into deciding. If the participant decides to take part in the study, he/she will be asked to date and sign the informed consent form. The potential participant will be requested to read through the consent form and the information sheet carefully and to clarify any misunderstandings before signing. Once the patient dates and sign the informed consent form, one of the investigators will also date and sign the aforementioned document. The participant will be given a copy of the signed document. The original signed informed consent form will be retained as part of the study records.

The formal consent of a participant, using the approved consent form, must be obtained before the participant is submitted to any study specific procedures.

All patients of whom intraoperative tissue and/or bone samples are collected and stored in the BioBank have signed the general consent „Einwilligungserklärung zur Weiterverwendung von biologischem Material und gesundheitsbezogenen Personendaten für die Forschung".

The collection of the general consents for further use of health-related personal data and biological material is a standard at UKB. It is not study-specific.

2.8 Participant privacy and confidentiality

The investigators affirm and uphold the principle of the participant's right to privacy and that they shall comply with applicable privacy laws. Especially, anonymity of the participants shall be guaranteed when presenting the data at scientific meetings or publishing them in scientific journals.

Individual subject medical information obtained as a result of this study is considered confidential and disclosure to third parties is prohibited. Subject confidentiality will be further ensured by utilising subject identification code numbers to correspond to treatment data in the computer files.

For data verification purposes, authorised representatives of the Sponsor (-Investigator), a competent authority (e.g. *Swissmedic*), or an ethics committee may require direct access to parts of the medical records relevant to the study, including participants' medical history.

2.9 Early termination of the study

The Sponsor-Investigators may terminate the study prematurely according to certain circumstances, for example:

- ethical concerns,
- insufficient participant recruitment,
- when the safety of the participants is doubtful or at risk, respectively,
- alterations in accepted clinical practice that make the continuation of a clinical trial unwise,
- early evidence of benefit or harm of the experimental intervention, e.g. based on interim analyses

2.10 Protocol amendments

Substantial amendments are only implemented after approval of the CEC.

Under emergency circumstances, deviations from the protocol to protect the rights, safety and well-being of human subjects may proceed without prior approval of the CEC. Such deviations shall be documented and reported to the CEC as soon as possible.

All non-substantial amendments are communicated to the CEC within the Annual Safety Report (ASR).

As substantial Amendments Count:

a) Changes, which affect security and health of the participants or their rights and duties.
b) Changes of the study protocol, due to new scientific findings, which affect study arrangement, study methods, objectives or statistical analysis.
c) Changes of the study location or inclusion of an additional study location.
d) Personnel changes, such as change of the sponsor-investigator.

3. BACKGROUND AND RATIONALE

3.1 Background Rationale

The duration of postoperative, systemic antibiotic therapy for implant-related orthopedic infections, with or without implant removal, is unknown[1-12].

Retrospective studies suggest that a maximum duration of 6 weeks is not inferior to longer administrations[1-5]; even if the infected implants are kept in place[1,3] or during a one-stage exchange[2]. Prospective-randomized trials (RCT) suggest that even shorter durations, such 3 or 4 weeks[6-10], are possible, when the implant is removed. Likewise, in prospective studies, 6 or 8 weeks of systemic antibiotics are not inferior[10-12] to the current 12 weeks during DAIR (debridement, antibiotic and implant retention)[1,3,12], or during the one-stage exchange[2,4]; except for one single RCT suggesting a better outcome for 12 weeks in the substrata of arthroplasty infections undergoing the DAIR procedure[13]. However, these RCTs concern selected branches of orthopedic surgery; especially prosthetic joint infections. We intend to expand these evaluations (with new RCTs) to all fields of orthopedic and hand surgery. The only exceptions would be spine surgery, for which a multicenter, separate RCT is already under way (SASI-trials)[10]. The second exception would be the treatment of implant-free diabetic foot infections, for which two distinct RCTs are already under way[7].

Finally, our study includes BioBanking. Instead of throwing away, we'll collect intraoperative infected tissues for ulterior studies not yet defined. Of note, BioBanking and participation in the clinical trial are exclusive among each other. Patients refusing to provide intraoperative tissue for BioBanking still have the choice to participate in the randomized study and *vice versa.*

3.2 Investigational Product and Indication

<u>Antibiotic agents used in the study</u>

The systemic antibiotic therapy is administered according current practice. Initially, antibiotic therapy is either empiric or targeted to the results of preoperative bone biopsy. After 2-6 days, antibiotic therapy becomes targeted to the pathogens identified in standard clinical cultures, and their antibiotic susceptibility profile. The choice of the agent, and its intravenous or oral administration route, is usually at discretion of the treating surgeon and/or infectious diseases consultant. Nevertheless, this study, and in order to achieve a minimal homogeneity, we established a list of "allowed antibiotics". The investigators must choose among them, unless the pathogens are special and need an antimicrobial agent outside of that list. We'll avoid topical antibiotic agents in this study population. The antibiotic agents used are officially indicated for orthopedic infections, including spine infections and available in Switzerland since decades. There will be no placebo and no dose investigations. Standard dosing will be applied and only modified according to the patient's co-morbidities, intolerances and weight.

The recommended systemic antibiotic list is as follows:

Allowed Antibiotic Agents (standard doses without renal insufficiency; to be adapted accordingly)

Antibiotic	Allowed Dosing Regimens	Allowed Total Daily Dose Range
Levofloxacin PO	750 mg q.24h or 500 mg q.12h	750 to 1500 mg
Ciprofloxacin PO	750 mg q.24h or 500 mg q.12h	750 to 1500 mg
Amoxicillin/clavulanate IV	1000/200 mg q.8h	2000/400 mg to 3000/600 mg
Cefuroxim IV	1500 mg q.8h	4500 mg
Ceftriaxon IV	2000 mg q.24 h	2000 mg
Co-trimoxazole PO	960 mg q.12h or q.8h	1920 mg to 2880 mg
Clindamycin PO	300 mg or 450 mg q.6h	1200 mg to 1800 mg
Doxycyclin PO	100 mg q.12h	200 mg
Minocyclin PO	100 mg q.12h	200 mg
Linezolid PO	600 mg q.12h	1200 mg
Metronidazole PO	500 mg q.8h	1500 mg
Vancomycin IV	15 mg/kg q.12h	according to serum through levels
Daptomycin IV	8 mg/kg/day	6-10 mg/kg/day
Cefepime IV	2000 mg q.12h	4000 mg
Imipenem IV	500 mg or 1000 mg or q.8h or q.6h	2000 mg to 3000 mg
Meropenem IV	2000 mg q.8h	1000 mg q.8h
Piperacillin/tazobactam IV	4000/500 mg q.8h	12000/15000 mg (12 g/1.5 g)

The daily doses will be standard as published in the compendium (www.compendium.ch), with adaptation according to patient's co-morbidities (e.g. renal insufficiency, obesity). We will not test special doses, placebos or new indications for antibiotic therapy. Only the duration of the therapy will be determined. All antibiotics are already on the Swiss market and approved by *Swissmedic*. Of note, anesthesiologists and surgeon are free to administer pre-operatively the routine antibiotic prophylaxis (cefuroxime, vancomycin, or clindamycin) for up to three consecutive doses, if they judge it clinically indicated.

3.2.1 Standard antibiotic prophylaxis used in the study

The standard prophylaxis used at UKB consists of one to three intravenous doses of cefuroxime 1.5 g intravenously; or 3 g if obesity; or vancomycin 1 g or clindamycin 600 mg if allergy. For this study, we do not determine the standard antibiotic prophylaxis or the necessity of this prophylaxis.

3.2.2 Definitions of an orthopedic infection

An orthopedic infection for this trial is the microbiological evidence of bacteria in at least two intraoperative tissue samples together with radiological (osteomyelitis, collections, inflammation) and/or clinical evidence of infection (pus, discharge, sinus tracts, rubor, calor, pain). Histological proof is facultative for this study. Implants are defined as any implants except for transient wires or fixator pins.

Remission of infection is the absence of clinical and/or radiological and/or laboratory signs of (former) infection after the minimal follow-up time of 1 year for implant -related surgery with implants left in place; or two months for implant-free surgery (including surgery when all implants have been removed). A clinical failure is any failure leading to an unplanned re-surgery within the follow-up time, including for persistent infection during antibiotic therapy, non-infectious reasons or new infections at the same site. A microbiological recurrence is a true recurrence of infection with the same pathogens at the same anatomical localization after the end of scheduled antibiotic therapy.

3.3 Clinical Evidence to Date

See chapter 3.1

3.4 Dose Rationale

See chapter 3.1

3.5 Explanation for choice of comparator

See chapter 3.1

3.6 Risks/Benefits of the study and of BioBanking

All patients can witness adverse events related to surgical procedures and antibiotic administrations, which however, are related to the therapy itself, and not to the specific study protocol.

For BioBanking specifically, a theoretical additional risk could be the detection of unknown pathologies, if there would be a further work-up of the intraoperative samples. In such a case, the investigators engage to inform the patient orally and by letter (unless the patient had explicitly expressed his/her wish not to be informed).

A theoretical risk could be a higher incidence of recurrences in the short antibiotic arm. In contrast, study patients and future patients with short antibiotic treatments might benefit from a shorter hospital stay, less AE, reduced costs, and more patients' satisfaction.

Pregnancy and breast-feeding

All antibiotics and surgeries, have no specific relations to pregnant or breast-feeding women and their children. Additionally, the study population is unlikely to reveal women at procreating age. Formally, pregnant and breast-feeding women are not excluded from this study. Concerning the choice of antibiotic drugs, the investigators will avoid agents that are not liberated for pregnant or breast-feeding women; according to the Swiss Compendium (www.compendium.ch). No study-specific pregnancy tests will be performed.

3.7 Justification of choice of study population

See chapter 3.1. No vulnerable participants are included.

4. STUDY OBJECTIVES

To reduce the post-debridement antibiotic duration in all sorts of orthopaedic in adult patients.

We evaluate if 6 weeks of systemic and targeted antibiotic therapy postoperatively is not inferior to 12 weeks (non-inferiority trial) in case of infections with a (new) implant (DAIR procedure or one-stage exchange), after a follow-up of 12 months. For orthopaedic infections with complete implant removal (or with external fixation only), the objective is the evaluation if 3 weeks of antibiotic therapy is not inferior to 6 weeks.

Secondary objectives are the assessments of differences regarding adverse events

Another objective is the assertion of infected tissue/bone for ulterior studies.

5. STUDY OUTCOMES

Primary outcome:

- Clinical remission related to the duration of total, postdebridement, antibiotic use
- Microbiological recurrence in relation to the total, postdebridement, antibiotic use

Secondary outcomes:

- Description of all clinical failures of any sort
- Adverse events in each study arm, and in relation to the antibiotics used
- Length of hospital stay in acute care surgery (without rehabilitation)
- BioBanking of infected tissue for ultimate laboratory studies

6. STUDY DESIGN

6.1 General study design and justification of design

We may prospectively collect the following key parameters and store intraoperative infected tissue samples (BioBanking).

These study-specific clinical variables are:

- Patient's characteristics: age, sex, known immune-suppression (diabetes mellitus, renal dialysis, cirrhosis, pregnancy, medicamentous immune-suppression, untreated HIV disease, agranulocytosis, active cancer), American Society of Anesthesiologists' (ASA)-Score

- Surgery specific baseline data: number and type of surgeries for the actual problem, agent, dose and duration of pre-surgical antibiotic therapy, local antibiotics used in the bone, cell count (if any), initial serum CRP level, presence of initial bacteremia.

- Anatomical localization of surgery, type of surgery, microbiological results, histology (facultative)

- Treatment and outcome: number of surgeries to treat infection, total duration of antibiotic therapy, duration, agent and dose of intravenous and oral antibiotic therapy, wound healing problems, presence and duration of vacuum-assisted negative pressure therapy, adverse events, clinical or and microbiological recurrence, date and reasons for re-hospitalization and re-treatment, follow-up data, fatalities

- Administrative data: total hospitalization length

- BioBanking of infected tissues at the Balgrist Campus

6.1.2 In case of refusal or withdrawal from the study

When a patient refuses to take part of the study, or is rejected by the investigators, his/her treatment will continue according to usual therapeutic standards and follow-ups. The patient can still donate his intraoperative tissue/bone for BioBanking.

If a patient's withdrawals his/her consent during the study period or up to one month after the last study visit, his/her information and results will be deleted from further analyses. Eventually, intraoperative samples for BioBanking will be destroyed.

6.1.3 Study duration

For the randomized study, we need 36 months; starting in Spring 2022 (CEC approval provided). The following Table highlights some key time events scheduled for the current study.

Timetable

P = Spring
S = Summer
A = Autumn
W = Winter

**Activity (Calendar year)**	**2022**				**2023**				**2024**				**2023**
	**P**	**S**	**A**	**W**	**P**	**S**	**A**	**W**	**P**	**S**	**A**	**W**	**P**
Ongoing recruitment of new sites	■	■	■	■	■	■	■	■	■	■			■
Clinical study			■	■	■	■	■	■	■	■	■		■
Database				■	■	■	■	■	■	■	■		■
Interim statistical analysis						■		■					
Final statistical analyses											■	■	
Writing-up of results and manuscript											■	■	

6.2 Methods of minimizing bias

The methods of minimising bias applied in our study are the randomization to add validity of the statistical tests used to demonstrate significance. The differences between intervention and control groups should behave like differences between two random samples from the population so that they can be compared to what would be expected in the population by chance.

6.2.1 Method of assignment to treatment/intervention (randomization, stratification)

After written informed consent has been given (up to 5d of surgery) participants will be randomized with a 1:1 ratio in either treatment group. Randomization is done by designated study staff drawing a sealed envelope containing a randomization card. Patients are informed about the assignment by the treating investigators.

6.2.2 Blinding procedures

There will be no blinding of patients, and no placebos.

6.3 Unblinding Procedures

n.a.

7. STUDY POPULATION

7.1 Eligibility criteria

Randomized Trials: All patients with orthopedic bone and implant infections. The study will start at UKB, but may be expanded to other centers in the future.

BioBanking: All patients with orthopedic bone and implant infections willing to participate in the Bio-Banking.

Inclusion criteria	• Age ≥ 18 years on admission<br>• Orthopedic bone and implant infections including sacral osteomyelitis and musculoskeletal grafts<br>• Intraoperative debridement with any surgical technique<br>• 12 months of scheduled follow-up from hospitalization<br>• Bacterial orthopedic infections of any nature, independently of implants or co-morbidities; according to clinical, laboratory, radiological, microbiological features of infection<br>• First or second episode of infection
Exclusion criteria	• Mycobacterial, fungal, nocardial, and *Actinomyces* infections<br>• Purely soft tissue infections<br>• Non-resected cancer in the infection site<br>• Purely intrasynovial infections (native joint septic arthritis)<br>• More than three debridements performed for infection<br>• Absence of at least one surgical intraoperative debridement<br>• Spine infections (investigated in another trial)[10]<br>• Diabetic foot infections (investigated in another trial)[7]<br>• Documented endocarditis according to the Duke criteria<br>• At least 2 prior infection episodes at the actual infection site<br>• Inability to understand the study procedure for language or cognitive reasons

7.2 Recruitment and screening

If infectious diseases physicians are consulted for post-surgery visits in patients having undergone surgery for orthopedic infections at UKB, the concerning patient will be screened. In case all inclusion and no exclusion criteria are met the patient will be informed about the study by one of the study investigators (see chapter 2.7).

As a routine procedure at UKB, all patients are asked to sign the general consents, including for intraoperative tissue samples, „Einwilligung zur Weiterverwendung nichtgenetischer gesundheitsbezogener Personendaten zu Forschungszwecken“ and „Einwilligungserklärung zur Weiterverwendung von biologischem Material und gesundheitsbezogenen Personendaten für die Forschung“.

If patients allow their data and biologic material to be used for research by signing the general consents described above they will be included in the BioBanking if applicable.

7.3 Assignment to study groups

Assignment to the study group (control versus intervention group) will be done with randomization cards or electronically.

7.4 Criteria for withdrawal / discontinuation of participants

All patients are free to withdraw from participation in this study at any time, for any reason, and without prejudice. A patient who withdraws consent by refusing to continue with study procedures/observations will be terminated from the study. The reason for withdrawal of consent should be clearly documented wherever possible. However, it is not required for patients to provide their reason.

The investigator should make every effort to address non-compliance issues and ensure that relevant study data are obtained from patients whenever possible.

To enable collection of follow-up data, the investigator may stop study treatment at any time without withdrawing the patient from the study (e.g. the patient experiences intolerable or unacceptable AEs possibly related to study treatment and where such treatment cannot be modified within the confines of the protocol).

On rare occasions, the investigator may terminate a patient from the study to protect the patient’s best interest e.g.

to protect them from excessive risk or risk with a demonstrated lack of benefits (serious side-effects) or to maintain the integrity of the data (when participates are not following study procedures or may be deliberately providing false information). The investigator must explain to the participant the reasons for the termination. If a patient is withdrawn before completing the study, the reason for withdrawal will be entered in the electronic case report form (eCRF). Whenever possible and reasonable, the evaluations that are required at the next scheduled visit will be performed at early termination.

8. STUDY INTERVENTION

8.1 Investigational Products (treatment / medical device)

Not applicable. All antibiotics are available on the Swiss market and approved by *Swissmedic*.

8.1.1 Intervention treatment

Short antibiotic arm:

The intervention group consists of 6 weeks of post-surgical targeted systemic antibiotic therapy (if implant).
This is 3 weeks without (remaining) implants.

8.1.2 Control Comparator

Long antibiotic treatment arm:

The control group consists of 12 weeks of post-surgical targeted systemic antibiotic therapy (if implant). This is 6 weeks without (remaining) implants.

8.1.3 Packaging, Labelling and Supply (re-supply)

According to *Swissmedic* guidelines. The Pharmacy of UKB supplies with all antibiotics. No study specific packing or labelling.

8.1.4 Storage Conditions

Medication is stored according to *Swissmedic* guidelines by the pharmacy of UKB.

The biobank will store intraoperative specimens in the Balgrist Campus at UKB. The UCAR team will establish and keep the identification key and list. The transport of intraoperative samples to the biobank will be performed as soon as possible either by the designated study nurse of UCAR or a designated collaborator of the biobank.

8.2 Administration of experimental and control interventions

8.2.1 Experimental Intervention

Short antibiotic arm:

Systemic antibiotic therapy applied according to standards published in the compendium (www.compendium.ch).

8.2.2 Control Intervention

Long intervention arm:

Systemic antibiotic therapy applied according to standards published in the compendium (www.compendium.ch).

8.3 Dose modifications

Dose modifications are provided according to international guidelines in patients with nephropathy and the publications of *Swissmedic* (www.compendium.ch).

8.4 Compliance with study intervention

No study specific surveillance of compliance (like diaries) with the prescribed antibiotic regimen will be implemented. In case of obvious incompliance (e.g. patient does not come to routine visits) the investigator can discontinue the patient from the study. His/her data remain in the study unless the patient declares explicitly that he/she wants to remove his/her data from the study. Idem for tissue/bone in case of BioBanking.

8.5 Data Collection and Follow-up for withdrawn participants

Withdrawn participants are instructed to continue therapy according to the corresponding control therapy protocol and given on whom to contact if there are any questions or concerns that arise after completing the study.

8.6 Trial specific preventive measures

None.

8.7 Concomitant Interventions (treatments)

Standard wound control for all patients will include eventual wound debridement (during hospitalization or at clinic visits and only if clinically indicated), regular wound care with dressing changes and eventual VAC use in selected cases upon surgical indication. We will avoid topical antibiotics

8.8 Study Drug Accountability

All drugs are commercial products and on stock at the UKB pharmacy. Drugs are ordered, stored and handled according to UKB pharmacy standards. No study specific drug accountability will be introduced.

8.9 Return or Destruction of Study Drug

Not applicable, commercial products.

9. STUDY ASSESSMENTS

9.1 Assessment of primary outcome

Clinical and microbiological outcome of treated infection at twelve months:

Judged by local wound healing (healed vs delayed wound healing according to Reference[4]). Remission is defined as the absence of clinical, anamnestic, radiologic or laboratory signs of former infection.

9.1.2 Assessment of other outcomes of interest

Secondary objectives are costs, duration of sick leave (if applicable), adverse events, length of hospital stay, mechanical sequelae and handicap after treatment of infection. We will note them during the study visits and obtain the necessary numbers from the administration services at UKB.

9.1.3 Assessment of safety outcomes

9.1.3.1 Adverse events

See chapter 10

9.1.3.2 Laboratory parameters

n/a. No study specific laboratory parameters will be assessed

9.1.3.3 Vital signs

n/a. No study specific vital sign measurements will be performed.

9.1.4 Assessments in participants who prematurely stop the study

Patients who withdraw consent or who, in the opinion of the investigator, are no longer able or eligible to participate in the study (including patients who require antibiotic therapy beyond Tx visit 5 (EOT) will be early terminated. Where possible, such patients will complete an early-termination visit to undergo all assessments applicable to the corresponding (or next) scheduled study visit. For these patients, we will record eventual adverse events, physical examination, laboratory parameters, vital signs during a follow-up period determined by clinical control, which is equally and usually up to one-year post intervention.

9.2 Procedures at each visit for both prospective-randomized studies

At enrollment (Day 1), the investigator will prescribe empiric antibiotic treatment based on instructions provided in the protocol and determine the most appropriate route of administration (oral or IV) according to the patient's condition. Patients will be randomized in the ratio 1:1 into the investigational group (short antibiotic treatment) and the control group (long antibiotic treatment)

Visit 1 - Enrollment (Day 1),

Visit 2 - Day 21 (+/- 5 days),

Visit 3 - Day 42 (+/- 5 days) (standard surgical orthopedic control in our setting)

Visit 4 - Day 84 (+/- 5 days).

End of treatment (EOT) visit - Day 21, 42 or Day 84 (+/- 5 days) (only if still receiving treatment after visit 2).

Test-of-cure (TOC) visits - 2 months (+/- 2 weeks) after EOT

Follow-up visit - 12 months (+/- 2 months) with implants; 6 months (+/- 2 months) without implants.

During the study visits, we assess the history, wounds, adverse events, and the functional status. We examine the patients according to the orthopedic standard, and add supplementary laboratory exams and radiology, only if clinically indicated.

9.2.1 Screening/Pre-procedure assessment/Visit 1

Information collected during routine pre-surgical consultation and during orthopaedic surgery is not study specific. This data will be used as general demographic information and medical history within the study in case of an orthopaedic infection and study participation.

Screening regarding a patient's suitability for the study will be done during post-surgery visits with involvement of infectious diseases physicians. If a patient appears to be eligible the following study-related procedures are performed:

1. Patient information and obtaining written informed consent.
2. Assign a study identification number.
3. Record/complete medical history and demographics, including the NRS (Nutrition) Score, if information is missing
4. Review inclusion/exclusion criteria.
5. Randomize the patient.

9.2.2 Visit 2, 3, 4, and End of Treatment

Outpatients will return to the clinic (assessments can be performed in the hospital for inpatients), where the following assessments will be performed:

1. Record any concomitant medications as well as any additional interventions required
2. Review clinical lab results from previous visit. If any results fall outside of the permitted inclusion criteria, the patient should be early terminated from the study and excluded from the study population.
3. Adaptation of (empirical) antibiotic therapy, if not done before.
4. Assess all adverse events of therapy.

9.2.3 Visit 5 (Test of Cure)

Every effort will be made to ensure that final efficacy assessments (i.e., primary outcome data) are available for all subjects. Outpatients should return to the clinic (assessments can be performed in the hospital for inpatients), where the following assessments will be performed:

1. Assess anamnestically all past adverse events of antibiotic therapy
2. Record clinical and microbiological recurrence and its treatment (if any)

9.2.4 Early Termination of Study Patients

Patients who withdraw consent or who, in the opinion of the investigator, are no longer able or eligible to participate in the study (including patients who require antibiotic therapy beyond EOT will be early terminated. Where possible, such patients will complete an early-termination visit to undergo all assessments applicable to the corresponding (or next) scheduled study visit.

10. SAFETY

10.1 Drug studies

During the entire duration of the study, all serious adverse events (SAEs) are collected, fully investigated and documented in source documents and case report forms (CRF). Study duration encompassed the time from when the participant signs the informed consent until the last protocol-specific procedure has been completed, including a safety follow-up period.

10.1.1 Treatment by specialists at UKB

All surgeries will be performed in the supervision and participation of an advanced and experienced surgeon. The antibiotic therapy is ordered and supervised by internists and infectious diseases physicians with therapeutic and academic experience orthopedic infection treatments. The current medications of the operated study patients, as well as possible interactions, will be controlled by the Head of Pharmacy UKB.

10.1.2 Definition and assessment of (serious) adverse events and other safety related events

An **Adverse Event (AE)** is any untoward medical occurrence in a patient or a clinical investigation participant administered a pharmaceutical product, and which does not necessarily have a causal relationship with the study procedure. An AE can therefore be any unfavourable and unintended sign (including an abnormal laboratory

finding), symptom, or disease temporally associated with the use of a medicinal (investigational) product, whether or not related to the medicinal (investigational) product.

A **Serious Adverse Event (SAE)** is classified as any untoward medical occurrence that:

- results in death,
- is life-threatening,
- requires in-patient hospitalization or prolongation of existing hospitalisation,
- results in persistent or significant disability/incapacity, or
- is a congenital anomaly/birth defect.

In addition, important medical events that may not be immediately life-threatening or result in death, or require hospitalisation, but may jeopardise the patient or may require intervention to prevent one of the other outcomes listed above should also usually be considered serious.

Examples of such events are intensive treatment in an emergency room or at home for allergic bronchospasm, blood dyscrasias or convulsions that do not result in hospitalization, or development of drug dependency or abuse.

SAEs should be followed until resolution or stabilisation. Participants with ongoing SAEs at study termination (including safety visit) will be further followed up until recovery or until stabilisation of the disease after termination.

Assessment of Causality

The investigators will make a causality assessment of the event to the study drug, based on the criteria listed in the ICH E2A guidelines:

Relationship	Description
Definitely	Temporal relationship<br>Improvement after dechallenge*<br>Recurrence after rechallenge<br>(or other proof of drug cause)
Probably	Temporal relationship<br>Improvement after dechallenge<br>No other cause evident
Possibly	Temporal relationship<br>Other cause possible
Unlikely	Any assessable reaction that does not fulfil the above conditions
Not related	Causal relationship can be ruled out
*Improvement after dechallenge only taken into consideration, if applicable to reaction	

Unexpected Adverse Drug Reaction

An “unexpected” adverse drug reaction is an adverse reaction, the nature or severity of which is not consistent with the applicable product information (e.g. Investigator’s Brochure for drugs that are not yet approved and Product Information for approved drugs, respectively).

Suspected Unexpected Serious Adverse Reactions (SUSARs)

The Sponsor-Investigator evaluates any SAE that has been reported regarding seriousness, causality and expectedness. If the event is related to the investigational product and is both serious and unexpected, it is classified as a SUSAR.

Assessment of Severity

This study uses a severity grading scale as described in the “Common Terminology Criteria for Adverse Events CTCAE Version 4.

10.1.3 Reporting of serious adverse events (SAE) and other safety related events

Reporting of SAEs

All SAEs must be reported immediately and within a maximum of <u>24 hours</u> to the Sponsor-Investigator of the study. The Sponsor-Investigator will re-evaluate the SAE and return the form to the site.

SAEs resulting in death are reported to the local Ethics Committee (via local Investigator) within 7 days.

Reporting of SUSARs

A SUSAR needs to be reported to the local Ethics Committee (local event via local Investigator) within 7 days, if the event is fatal, or within 15 days (all other events).

Reporting of Safety Signals

All suspected new risks and relevant new aspects of known adverse reactions that require safety-related measures, i.e. so-called safety signals, will be reported to the Sponsor-Investigator within 24 hours. The Sponsor-Investigator will report the safety signals within 7 days to the local Ethics Committee (local event via local Investigator).

Reporting and Handling of Pregnancies

This study, all antibiotics and therapeutic surgeries, have no specific relation to pregnant or breast-feeding women and their children. The study population is likely not to reveal pregnant women. Formally, pregnant and breast-feeding women thus are not excluded from this cohort and its side studies.

Any pregnancy during the treatment phase of the study and within 30 days after discontinuation of study medication will be reported to the Sponsor-Investigator within 24 hours. The course and outcome of the pregnancy will be followed up carefully, and any abnormal outcome regarding the mother or the child should be documented and reported.

Periodic reporting of safety

An annual safety report on the participant is submitted once a year to the local Ethics Committee via the Lead Investigator. We, moreover, will perform statistical interim (futility) analysis on an annual basis.

10.1.4 Follow up of (Serious) Adverse Events

Participants terminating the study (either regularly or prematurely) with reported ongoing SAE, or any ongoing AEs of laboratory values or of vital signs being beyond the alert limit will return for a follow-up investigation. This visit will take place up to 30 days after terminating the treatment period. Follow-up information on the outcome will be recorded on the respective AE page in the CRF/eCRF. All other information has to be documented in the source documents. Source data have to be available upon request.

In case of participants lost to follow-up, efforts will be made and documented to contact the participant to encourage him/her to continue study participation as scheduled. In case of minor AE, a telephone call to the participants is acceptable.

All new SAE or pregnancies that the investigators will be notified of within 30 days after discontinuation of study medication will be reported in appropriate report forms and in the CRF/eCRF if required.

Follow-up investigations may also be necessary according to the investigator's medical judgment even if the participant has no AE at the end of the study. However, information related to these investigations does not have to be documented in the CRF/eCRF, but must be noted in the source documents.

11. STATISTICAL METHODS

11.1 Main hypotheses

A six week's course of targeted systemic antibiotic therapy after the first debridement for an orthopedic infection is not inferior to twelve weeks (if there are implants left in place). Likewise, a three week's course of targeted systemic antibiotic therapy after the first debridement for orthopedic infection is not inferior to six weeks (if there are no implants).

11.2 Determination of Sample Size

The RCTs are non-inferiority trials. Remissions (at the first therapeutic approach) are set at 94% (6% recurrences in both arms). The maximum acceptable difference (unidirectional lower margin with binary-outcome categorical variables) is arbitrarily fixed at 10% regarding the primary outcome remission. Assuming a risk of alpha at 0.05 and a power of 80%, it will be necessary to recruit 70 patients in each antibiotic duration arm (short or long). Together with the distinction of the RCT into implant-related and implant-free bone infections, we would finally need 2 x 2 x 70 episodes, equaling a total of 280 episodes within three years; including for all strata of orthopedic infections. With very few anticipated dropouts, we would probably need 300 episodes.

11.3 Planned Analyses

All analyses will be performed for the entire study population. In a second step, all analyses will be separately performed according substrata of patients basing on the orthopaedic speciality. We will use descriptive statistics, perform group comparisons (using the Pearson-χ^2-tst, the Fischer-exact-test, or the Wilcoxon-ranksum-test, as appropriate. We will also perform separate multivariate analysis using a Cox regression model targeting the primary outcome variables[14]. For assessment the formal non-inferiority requirement (regarding the primary outcome “clinical remission”), we will compute with a unidirectional p-value limit of 0.025. Formal non-inferiority assessment for "microbiological recurrence" will not be necessary because of the few numbers of events anticipated. Likewise, we do not predefine a non-inferiority margin for secondary outcomes such adverse events.

11.3.1 Interim analyses and early termination

We will perform two interim analyses after one and two years following the inclusion of the first patient. If group comparison between the corresponding short and long antibiotic course are striking and statistically significant in terms of any study objectives[15], the independent Data Monitoring committee will decide upon the interruption and early termination of the study (or, alternatively, to continue the study only in one substratum; e.g. patients with or without infected orthopedic implants). Otherwise, the study continues until the next interim analysis. Of note, the Data Monitoring committee has the right to call on a premature, additional interim analysis.

If the crude group comparison analysis is not sufficiently meaningful, we will perform a futility analysis to check if the expected statistical power for the final analysis will not be unacceptable (more than 30%). If it is lower than 30%, we will consider the trial will not be able to demonstrate the result, and the recruitment will be no more ethical. The most frequents conditional power evaluated under the current trend (i.e. using the information from the collected data) will be assessed.[35,36] It was demonstrated that futility analyses decrease the statistical power of the final analysis in superiority trials, but in our knowledge, this topic was not explored for non-inferiority trials. To balance (at least partially) this loss of power, it is planned to recruit 50 supplementary patients per arm.

11.3.2 Final analyses

The intent-to-treat (ITT) population will consist of all randomized patients. Patients will be analysed according to treatment group assignment regardless of whether the patient receives any study treatment. Patient disposition and baseline characteristics will be based on the ITT population.

The per-protocol (PP) population will consist of all randomized patients who complete the study (or who are otherwise defined as a treatment failure) and who have not deviated significantly from the protocol. All efficacy analyses will be repeated using the PP population. Any analysis involving microbiological assessments will exclude patients without an assigned baseline pathogen.

For the primary outcome parameter, univariable and multivariable results will be computed using a Cox regression analysis. Variables with a p value ≤0.2 in univariate analysis will be included in a stepwise forward selection process for multivariate analysis. Key variables will be checked for co-linearity and interaction. The number of variables in the final model is limited to the ratio of 1 variable to 5 to 8 outcome events.[37]

11.4 Handling of missing data and drop-outs

Missing data will lead to patient dropout of the study. Drop-outs will be reported in the patients & methods section of the publication, drop-out data will archived for a minimum of 10 years after study termination or in case of premature termination of the clinical trial.

12. QUALITY ASSURANCE AND CONTROL

The Sponsor-Investigator will implement and maintain quality assurance and quality control systems with written SOPs and Working Instructions to ensure that trials are conducted, and data are generated, documented (record), and reported in compliance with the protocol, GCP, and applicable regulatory requirement(s). Monitoring and Audits will be conducted during the course of the study for quality assurance purposes.

12.1 Data handling and record keeping / archiving

Data is exclusively stored using the secured REDCap® electronic data capture tool. The PI is responsible for collection of data and possesses the screening log, where confidentiality is ensured by using participants’ ID. Study IDs are distributed by REDCap® automatically in ascending order. Access authorization via Log-in (User) and password will be given by the PI to people on the staff list involved in the study as necessary. For this reason, data cannot be changed by non-authorized people. REDCap® documents every relevant processing step to ensure traceability with registration software and is secured daily via backups. All transaction logs between performing of two backups will be secured for one week while every study data in REDCap® will be secured for an unconfined time, at least for 10 years. Its data base server is allocated in highly modern rooms in Rümlang ZH and Altstetten ZH with protection of access. Collected data of this study is visible for inspection of independent ethic committee and authorities.

When the study is terminated, data will be stored in the same system. Data can only be accessed by defined persons that have contributed to the project. Source Data are going to be stored in the institutions PACS and

KISIM system according to the institutional standard at the UKB.

12.1.1 Case Report Forms

Electronic case report forms (eCRF) will be used, one for each enrolled study participant, to be filled in with all relevant data pertaining to the participant during the study. All participants who either entered the study or were considered not-eligible or were eligible but not enrolled into the study have to be documented on a screening log. The participation of each study participant will be documented on the Enrolment Log. For data and query management, monitoring, reporting and coding an internet-based secure data base REDcap® developed in agreement to the Good Clinical Practice (GCP) guidelines will be used. It is the responsibility of the PI to assure that all data in the course of the study will be entered completely and correctly in the respective data base. Corrections in the eCRF may only be done by the investigator or by other authorized persons. In case of corrections the original data entries will be archived in the system and can be made visible. For all data entries and corrections date, time of day and person who is performing the entries will be generated automatically. Documented medical histories and narrative statements relative to the participant's progress during the study will be maintained. These records will also include the following: originals or copies of laboratory and other medical test results (e.g. ECGs, etc.) which must be kept on file with the individual participant's eCRF. The investigators assure to perform a complete and accurate documentation of the participant data in the eCRF.

12.1.2 Specification of source documents

Source data will be available at the site to document the existence of the study participants and substantiate the integrity of study data collected. Source data will include the original documents relating to the study, as well as the medical treatment and medical history of the participant.

The following information (at least but not limited to) will be included in the source documents:

- Demographic data (age, sex).
- Inclusion and Exclusion Criteria details.
- Participation in study and signed and dated Informed Consent Forms.
- Visit dates.
- Medical history and physical examination details.
- Key efficacy and safety data (as specified in the protocol).
- SAEs, AEs and concomitant medication.
- Results of relevant examinations.
- Laboratory printouts.
- Reason for premature discontinuation.
- Randomization number.

Source data will always be kept with the regular patient file.

The following documents are also considered source data, including but not limited to:

- SAE worksheets.
- Nurse records, records of clinical coordinators.
- Medical records from other department(s), or other hospital(s), or discharge letters and correspondence with other departments/hospitals, if participant visited any during the study period and the post study period.

12.1.3 Record keeping / archiving

All study data must be archived for a minimum of 10 years after study termination or premature termination of the clinical trial. Data are stored using the proprietary hospital information system and REDCap electronic data capture tool hosted at the UKB.

12.1.4 BioBanking

Intraoperative tissue and bone specimens will be stored at the BioBankling facility at BioCampus Balgrist, approximatively 50 meters near the operation theatres. The specimens will be encoded. During and after the study, the key assigning the samples to the patient will be stocked in the offices of the Head of BioBanking and the Head of UCAR team; not accessible for any study nurse, clinicians or administrative persons.

12.2 Data Management System, access and back-up

Subject-related data will be stored in the research electronic data capture software REDCap. Back up will be kept on a hard drive belonging to the PI and later on stored in the archives of the UKB, as mentioned above. The PI and the co-investigators are responsible for data recording. The PI will grant the relevant personnel user rights to view and/or edit data entries by password as applicable. All edits will be automatically documented in the change history log.

12.2.1 Analysis and archiving

For data analysis, subject-related data from REDCap will be exported and analyzed in statistics software (IBM –

SPSS and/or STATA, Version 14, College Station, USA). Before data export, all patient identifiers will be removed. All eCRF data will be stored for a minimum of 20 years.

12.3 Monitoring

Regular monitoring visits at the investigator's site prior to the start and during the course of the study will help to follow up the progress of the clinical study, to assure utmost accuracy of the data and to detect possible errors at an early time point. The Sponsor-Investigator organizes professional independent monitoring for the study.

All original data including all patient files, progress notes and copies of laboratory and medical test results must be available for monitoring. The monitor will review all or a part of the eCRFs and written informed consents. The accuracy of the data will be verified by reviewing the above referenced documents.

One monitoring visit at the investigator's site prior to the start and twice during the course of the study will be organised by the Sponsor-Investigator. Furthermore, there will be a close-out visit at the study end. During the monitoring, all documents including source data/documents will be accessible for the monitor and all questions will be answered.

**Study period**	**Time**	**Monitoring**
Before study	Spring 2022	Monitoring will be informed about study conduct concerning data sampling and safety reporting.<br>Monitor controls if<br>• Documents are approved<br>• Documents are at site<br>• Investigators are familiar with study protocol and safety reporting<br>• Investigators know their duties and responsibilities
During Study	Spring-Sommer 2023	All subjects: SDV for existence and informed consent<br>First trial participant and at least 10% of trial participants recruited at the time of the visit, as far as available: eligibility, primary end-point, SAEs
Study end	December 2024	Control for completeness of source data

12.4 Audits and Inspections

A quality assurance audit/inspection of this study may be conducted by the competent authority or CEC, respectively. The quality assurance auditor/inspector will have access to all medical records, the investigator's study related files and correspondence, and the informed consent documentation that is relevant to this clinical study. The investigator will allow the persons being responsible for the audit or the inspection to have access to the source data/ documents and to answer any questions arising. All involved parties will keep the patient data strictly confidential.

12.5 Confidentiality, Data Protection

Direct access to source data may be granted in the case of monitoring, audit or inspections. All personnel must treat patient data as confidential. As far as possible, encoded data will be used. Only persons listed on the staff list have access to the source data.

12.6 Storage of biological material and related health data

All health-related patient data will be stored and archived in the data capture software REDCap. Patient-source data will be registered using subject identifiers. After full data analysis, all subject identifiers will be erased. Patient-source data may still be saved in the patient's medical record. Collection, disclosure, storage of patient-related data is carried out in accordance with Swiss data protection regulations and the Human Research Act. A requirement is the informed consent of every subject prior to inclusion in the clinical trial. The BioBank will store the intraoperative tissue samples in accordance with laboratory guidelines as standard.

13. PUBLICATION AND DISSEMINATION POLICY

After the statistical analysis of this trial the sponsor will make every endeavour to publish the data in (a) medical journal(s), to be able to communicate the results to healthcare professionals, the public and other relevant groups. All participants will be sent a free copy of the published article. There will not be any publication restriction and we plan to sort at least three major publications. We will also present preliminary results in national, regional, and international scientific meetings.

All investigators indicated in this protocol, and eventually additional colleagues participating in the future, will be co-authors of this study according to their individual contributions. The main study nurse, all Sponsors and Principal Investigators will participate in all publications. In selected publications, members of the corresponding orthopaedic teams will participate as co-authors and depending on their investment into the study.

14. FUNDING AND SUPPORT

We start with a funding by the Research Funds of UKB. This initial internal financing of UKB will be 10’000 Swiss Francs. In addition, the investigators will demand for grants to obtain more study funding. Of note, most investigators are employees of the UKB and perform this study in proper academic interest. There are no material costs as the infrastructure to conduct such RCT is already available in UKB, and running[7,10].

15. INSURANCE

The standard Balgrist research insurance is applicable.

Insurance police Nr. 14.050.565 Winterthur Versicherung.

Any damage developed in relation to study participation is covered by this insurance. So as not to forfeit their insurance cover, the participants themselves must strictly follow the instructions of the study personnel. Participants must not be involved in any other medical treatment without permission of the principal investigator (emergency excluded). Medical emergency treatment must be reported immediately to the investigator. The investigator must also be informed instantly, in the event of health problems or other damages during or after the course of study treatment. The investigator will allow delegates of the insurance company to have access to the source data/documents as necessary to clarify a case of damage related to study participation. All involved parties will keep the patient data strictly confidential. A copy of the insurance certificate will be placed in the Investigator’s Site File and the trial master file.

16. REFERENCES

1) Chaussade H, Uçkay I, Vuagnat A, et al. Antibiotic therapy duration for prosthetic joint infections treated by Debridement and Implant Retention (DAIR): Similar long-term remission for 6 weeks as compared to 12 weeks. Int J Infect Dis 2017;63:37-42.

2) Bernard L, Legout L, Zürcher-Pfund L, et al. Six weeks of antibiotic treatment is sufficient following surgery for septic arthroplasty. J Infect. 2010;61(2):125-32.

3) Hirsiger S, Betz M, Stafylakis D, et al. The Benefice of Mobile Parts' Exchange in the Management of Infected Total Joint Arthroplasties with Prosthesis Retention (DAIR Procedure). J Clin Med. 2019;8(2):226.

4) Farhad R, Roger PM, Albert C, et al. Six weeks antibiotic therapy for all bone infections: results of a cohort study. Eur J Clin Microbiol Infect Dis. 2010;29(2):217-22.

5) Puhto AP, Puhto T, Syrjala H. Short-course antibiotics for prosthetic joint infections treated with prosthesis retention. Clin Microbiol Infect. 2012;18:1143-8.

6) Gariani K, Pham TT, Kressmann B, et al. Three versus six weeks of antibiotic therapy for diabetic foot osteomyelitis: A prospective, randomized, non-inferiority pilot trial. Clin Infect Dis. 2020 Nov 26;1758.

7) Waibel F, Berli M, Catanzaro S, et al. Optimization of the antibiotic management of diabetic foot infections: protocol for two randomized controlled trials. Trials. 2020;21(1):54.

8) Benkabouche M, Racloz G, Spechbach H, et al. Four versus six weeks of antibiotic therapy for osteoarticular infections after implant removal: a randomized trial. J Antimicrob Chemother. 2019;74(8):2394-9.

9) Rod-Fleury T, Dunkel N, Assal M, et al. Duration of post-surgical antibiotic therapy for adult chronic osteomyelitis: a single-centre experience. Int Orthop. 2011;35(11):1725-31.

10) Betz M, Uçkay I, Schüpbach R, et al. Short postsurgical antibiotic therapy for spinal infections: protocol of prospective, randomized, unblinded, noninferiority trials (SASI trials). Trials. 2020 Feb 6;21(1):144.

11) Argenson JA, Arndt M, Babis G, et al. Hip and Knee Section, Treatment, Debridement and Retention of Implant: Proceedings of International Consensus on Orthopedic Infections. J Arthroplasty. 2019 Feb;34(2S):399-419.

12) Lora-Tamayo J, Euba G, Cobo J, et al. Short- versus long-duration levofloxacin plus rifampicin for acute staphylococcal prosthetic joint infection managed with implant retention: a randomised clinical trial. Int J Antimicrob Agents. 2016;48(3):310-6.

13) Bernard L, Arvieux C, Brunschweiler B, et al. Antibiotic Therapy for 6 or 12 Weeks for Prosthetic Joint Infection. N Engl J Med. 2021 27;384(21):1991-2001.35. Lachin JM. A review of methods for futility stopping based on conditional power. Statist Med. 2005;24:2747-64.

14) Vittinghoff E, McCulloch CE. Relaxing the rule of ten events per variable in logistic and Cox regression. Am J Epidemiol. 2007;165:710-18.

15) Snapinn S, Chen MG, Jiang Q, Koutsoukos T. Assessment of futility in clinical trials. Pharmaceutical Statistics. 2006;5:273-81.
