# Supplementary material for: Study protocol: short against long antibiotic therapy for infected orthopedic sites — the randomized-controlled SALATIO trials
Source: Trials. 2023 Feb 18;24:117. doi: 10.1186/s13063-023-07141-2 (PMC9938993; doi:10.1186/s13063-023-07141-2)
Supplement: Supplementary file 2 — Additional file 2: Supplementary File 2. Consent Form in English language. SALATIO Trials. [file 13063_2023_7141_MOESM2_ESM.docx]

**Patient information / Consent Form**

**Optimization of postsurgical antibiotic duration in orthopedic bones and implant infections**

*Original Title: Short Against Long Antibiotic Therapy for Infected Orthopedic Sites*

*- the randomized-controlled SALATIO Trials*

This study is organized by:

Prof. Dr. med. Ilker Uçkay, Balgrist University Hospital

PD Dr. med. Patrick Zingg, PD Dr. med. Stephan Wirth, PD Dr. Björn Zörner

Dear Patient, Dear Patient

You are treated for an orthopedic (paraplegic) bone and/or implant infection. In addition to the surgical intervention, you need antibiotics. We would like to ask you whether you would participate in a clinical trial regarding the duration of postoperative antibiotic prescription; and not the choice of antibiotics.

**1. Aim of the study**

The aim of the study is to determine the optimal duration of antibiotic therapy for operated orthopedic infections. We distinct between distinction between the cases with and without the presence of an infected implant, and record the success of antibiotic treatment and its eventual side effects.

**2. Selection**

All adult persons who are treated for an orthopedic bone and /or implant infection, and who are treated by surgery and antibiotic therapy, can participate. You must also be at least 18 years old. Persons with concomitant heart valve infections, actual in the operating area, or who participate in similar studies in the spine surgery, and for a diabetic foot osteomyelitis, cannot participate.

**3. Allgemeine Information**

Orthopedic infections are a high burden for patients. There are few scientific studies to optimize the antibiotic therapy of these infections. Surgeons and physicians usually prescribe antibiotics for 4, 6 or 12 weeks, even if the infected tissue is removed (debridement). Previous studies have shown no advantage of extending antibiotic therapy to more than 6 weeks; with only one exception of a single study from France. Studies tend to suggest that 6 weeks is just as good as longer antibiotic durations, and that a much shorter duration may be preferable; especially since side effects on antibiotics are common.

In this prospective (forward-looking), randomized (participating patients are randomly assigned to different groups) study, patients receive a targeted antibiotic therapy of 6 or 12 weeks (if an implant is still present), or for 3 or 6 weeks (if no implant is present). The choice of the antibiotic agent (drug) depends on the pathogens and is not subject of this study. We only use antibiotics that have been approved for these indications in Switzerland since several decades. We do not test placebo, new dosages/antibiotics, or new molecules.

The surgery itself is not part of the study and is performed by your surgeon. It may be necessary to re-operate again if there is a clinical necessity. Likewise, the antibiotic prophylaxis that you will receive before and after surgery, is a standard procedure for every intervention.

Additionally, if you agree, we will freeze tissue that will be removed during surgery. This tissue is usually thrown away after the analysis. However, we would like to use it for possible future research projects. We also collect some minimal data about your health from your medical history. This will only happen if you give us a separate consent at the end of this document and/or have signed the general consensus of the hospital.

The study is expected to last a total of 3 years. For you as a participant, the study participation stops at 6 to 12 months after completion of therapy (depending on whether an implant is infected). These are part of our usual follow-ups after orthopedic surgery on the bone or implants. This study does not generate any additional costs, appointments or clarifications for you, or for your health insurance.

In total, we will enroll approximately 280 patients in the study. We are conducting this study in accordance with Swiss law. In addition, we follow all internationally recognized guidelines. The cantonal Ethics Committee in Zurich reviewed and approved the study.

**4. Procedure**

If you agree to participate in this study, you will be randomly assigned to an antibiotic duration (6 or 12 weeks if the implant is present; or 3 versus 6 weeks without an implant). The probability is the same. Before and/or during the operation for infection treatment, we take samples for microbiological identification. The study begins immediately after the first operation for the infection. The antibiotic agents will be given to you, as prescribed by your attending physician. You may be excluded from the study in your best interest. This can happen if, for example, you need a longer course of antibiotic therapy, or if you no longer qualify for participation from a medical point of view.

**5. Benefits**

You may benefit from shorter antibiotic therapy (for example, possibly fewer side effects). The results can be important to optimize the duration of antibiotic therapy for future patients. However, it may also be that you do not have any special advantages from participating.

**6. Rights**

They participate voluntarily. If you do not want to participate, you do not have to justify. You may ask questions about the study procedures and your individual results at any time. To do so, please contact the person named at the end of this information. If you do not participate in this study, you do not have to expect any disadvantages for your further medical care. The same applies if you revoke your consent to participate in the study at a later date.

**7. Obligations**

As a participant, it is important that you

- adhere to the recommendations and prescriptions of your attending physician.
- Inform your physician about the course of the disease and report new symptoms, new complaints; or any changes in your condition
- inform the investigator about other, concomitant treatments and therapies and changes in your medication.

**8. Risks and burdens for participants**

The patients with shorter antibiotic treatments could theoretically have more recurrences. Patients with longer antibiotic treatments may experience increased side effects (skin rash, nausea, allergy) that are unpredictable. In the event of a relapse or insufficient response, antibiotic therapy will be resumed, or continued, so that your protection guaranteed. If it is necessary to continue the antibiotic therapy for a longer period than scheduled, we will accompany you during that prolonged treatment and perform medical controls. In contrast, data and any new results that occur during further treatment are no longer evaluated.

**9. Other treatment options**

You do not have to participate in this study. If you do not participate, the duration of your antibiotic therapy will be determined according to the current "standards" of Balgrist University Hospital. These standards are 6 weeks of antibiotic therapy (intravenous or oral) for infections without implants and a maximum of 12 weeks for infections with implants.

**10. Findings from the study**

The investigator will inform you during the study of any new findings that may affect the benefit of the study or your safety; and thus, your consent to participate in the study. You will receive the information orally or written. You will be informed of incidental findings that can help prevent, detect and treat existing or anticipated future diseases. If you do not wish to be informed, please inform your investigator.

**11. Confidentiality of data and samples**

We will collect a minimal amount of your personal and medical data for this study. Very few professionals will see your unencrypted data, and only to perform tasks within the scope of the study. When data is collected for study purposes, the data is encrypted. Encryption means that all reference data that could identify you (name, date of birth) is deleted: and replaced with a key. The key list always remains at Balgrist University Hospital. Those people who do not know the key can therefore not draw any conclusions about your person. In the case of a publication, the individual data are not traceable. We will not publish your name anywhere, in any report, publication, print or in the Internet. Raw data for the publication of scientific reports in scientific journal will be always in encrypted form.

This study may be reviewed by an Ethics Committee, *Swissmedic* or the institution that initiated the study. They all ensure that the research rules are adhered to and that your safety is not compromised. The study leader may need to disclose your personal and medical information upon explicit demand of the regulatory authorities.

**12. Withdrawal**

You can stop and withdraw from the study at any time. The data collected until then is still evaluated in the encrypted form. After the publication of the study, your data will be completely anonymized. Your key assignment will be destroyed so that no one can know that the data originally stem from you.

**13. Compensation for participants**

You will not receive any remuneration for participating in this clinical trial. You or your health insurance company do not incur any costs because of study participation.

**14. Liability**

If you suffer damage as a result of the study itself, the institution responsible for conducting the study is liable. The procedure is regulated by law. Balgrist University Hospital has taken an insurance with AXA Winterthur Versicherungen AG, General-Guisan-Strasse 40, 8400 Winterthur, in order to be able to pay for liability in the event of damage. In the case of damage due to an approved remedy used in accordance with the medical standard, or that would have occurred if a conventional therapy had been used, the same liability rules apply. If you have suffered damage, please contact the persons indicated below or the insurance company mentioned above.

**15. Financing of the study**

This study is paid by the scientific fund of Balgrist University Hospital and possibly by other sources, such as research funds from foundations and authorities.

**16. Contact person**

In case of ambiguities, emergencies, unexpected situations, or adverse events that occur during or after the study is completed, you can always contact the following persons:

| PD Dr. med. Stephan Wirth  Head of Foot Team  Forchstrasse 340  8008 Zurich  Phone 044 386 11 11  E-Mail: [fuss@balgrist.ch](mailto:fuss@balgrist.ch) | PD Dr. Björn Zörner  Chief Physician Polyclinic NDT  Forchstrasse 340  8008 Zurich  Phone 044 386 11 11  E-Mail: [zfp@balgrist.ch](mailto:zfp@balgrist.ch) |
| --- | --- |
| PD Dr. med. Patrick Zingg  Head of Hip Team  Balgrist University Hospital  Forchstrasse 340  8008 Zurich  Phone 044 386 11 11  E-Mail: [huefte@balgrist.ch](mailto:huefte@balgrist.ch) | Prof. Dr. med. Ilker Uçkay  Infectiology, Head of Clinical Research  Balgrist University Hospital  Forchstrasse 340  8008 Zurich  Phone 044 386 11 11  E-Mail: [ilker.uckay@balgrist.ch](mailto:ilker.uckay@balgrist.ch) |

**Consent**

**Written declaration of consent to participate in a study project**

Please read this form carefully. Please ask if you do not understand or do not want to know something. Your written consent is required for participation.

| **Title of the study:** | Optimization of the postsurgical antibiotic duration in othopedic bones and implant infections  *Original Title: Short Against Long Antibiotic Therapy for Infected Orthopedic Sites*  *- SALATIO Trials* |
| --- | --- |
| **Responsible institution:** | Balgrist University Hospital |
| **Place of implementation**: | Balgrist University Hospital  Forchstrasse 340  CH-8008 Zurich |
| **Responsible investigator at the study site:** | Prof. Dr. med. Ilker Uçkay |
| **Participant:** Surname and first name. Date of birth: | female male |

- I was informed orally and by written information, by the investigator about the purpose, the course of the study, possible advantages and disadvantages as well as risks.
- I participate voluntarily in this project and accept the content of the written information provided on the above-mentioned project. I had enough time to make my decision.
- My questions related to participation in this project have been answered. I keep the written information and receive a copy of my written declaration of consent.
- I am informed that an insurance covers the damage to health, if such occurs in the context of the study.
- I agree that the responsible experts of the project management/client of the study and the Ethics Committee responsible for this project may inspect my unencrypted data for examination and control purposes, but in strict compliance with confidentiality.
- In the event of study results or incidental findings that would directly affect my health, I am informed. If I do not wish this, I will inform my investigator.
- I know that my health and personal data can only be shared in encrypted form for research purposes and only for this study project.
- I agree that, if I continue to be treated outside the study site, the investigator/project leader may contact the treating physicians to request follow-up data relevant to the study.
- I participate in this study voluntarily. I can withdraw from participation at any time and without giving reasons, without having any disadvantages in further medical care. The data and samples collected until then will still be used for evaluation of the study.
- I am aware that during the study, the requirements and restrictions in the patient information must be observed. In the interest of my health, the investigator may exclude me from the study.

| Place, Date | Signature of the participant |
| --- | --- |

**Confirmation of the investigator: I** hereby confirm that I have explained the nature, significance and scope of the study to this participant. I agree to comply with all obligations related to this study in accordance with applicable law. If, at any time during the conduct of the study, I become aware of aspects that could influence the participant's willingness to participate in the study, I will inform him/her immediately.

| Place, Date | Surname and first name of Investigator |
| --- | --- |
|  | Signature Investigator |

**Declaration of consent for further use of (genetic) data and biological material in encrypted form**

**(for the further use of data and samples from THIS study)**

| **Participant:** Surname and first name in block capitals: Date of birth: | female  male |
| --- | --- |

I allow my (genetic) data and samples from this study to be used for medical research. This means that the samples may be stored in a biobank and used for future, as yet undefined research projects for an indefinite period of time. This consent is valid indefinitely.

I decide voluntarily and can revoke this decision at any time. If I resign, my (genetic) data will be anonymized and my samples destroyed. I only inform my investigator and do not have to justify this decision.

I understood that the data and samples are encrypted and the bowl is kept safe. The data and samples can be sent to other data and biobanks in Switzerland and abroad for analysis if they comply with the same standards as in Switzerland. All legal requirements for data protection are complied with.

Normally, all data and samples are evaluated as a whole and the results are published in summary. If there is an important result for my health, it is possible that I will be contacted via my investigator. If I do not wish this, I will inform my investigator.

If results from the data and samples are commercialized, I have no claim to a share of the commercial use.

| Place, Date | Signature of participant (or legal representative) |
| --- | --- |

**Confirmation of the investigator: I** hereby confirm that I have explained to this participant the nature, meaning and scope of the further use of samples and/or genetic data.

| Place, Date | Surname and first name of the informing investigator in block capitals  Signature of the investigator |
| --- | --- |
