# Supplementary material for: Study protocol: short against long antibiotic therapy for infected orthopedic sites — the randomized-controlled SALATIO trials
Source: Trials. 2023 Feb 18;24:117. doi: 10.1186/s13063-023-07141-2 (PMC9938993; doi:10.1186/s13063-023-07141-2)
Supplement: Supplementary file 3 — Additional file 3: Supplementary File 3. Internal Document and Proof of Funding. [file 13063_2023_7141_MOESM3_ESM.pdf]

**Studien-Nr** (nicht ausfüllen)	Wissenschaftsnummer W956; Balgrist University Hospital

**Physicians / surgeons**	**Prof. Dr. med. Ilker UCKAY**	
Name of Trial	**Short Against Long Antibiotic Therapy for Infected Orthopedic Sites - SALATIO trials**	

**Submission:**		**Target Journal**	**High Impact**

**Funding**<br>- Research Personal<br>- Ethical Comitttee (ca. 1000-1500.-)	Start Funding:	**CHF 8000 (eight thousand Swiss Francs)**
	Financial source (internal; Balgrist University Hospital)	X Wi-Konto Orthopädie<br>❍ Wi-Konto Radiologie<br>❍ Wi-Konto Anästhesie<br>❍ Wi-Konto Rheumatologie

**1. OK UCKI (UCAR)**	Date:29.4.22		Visum: I. Uçkay	
**Prof. Ilker Uckay**	Approved	X	Not approved	❍
